# Dyadic Coping in Couples Facing Chronic Physical Illness: A Systematic Review

**DOI:** 10.3389/fpsyg.2021.722740

**Published:** 2021-10-25

**Authors:** Katharina Weitkamp, Fabienne Feger, Selina A. Landolt, Michelle Roth, Guy Bodenmann

**Affiliations:** ^1^Clinical Psychology Children/Adolescents and Couples/Families, University of Zurich, Zurich, Switzerland; ^2^ZHAW Zurich University of Applied Science, Zurich, Switzerland

**Keywords:** dyadic coping, couple coping, chronic physical illness, intimate relationship, review

## Abstract

**Objective:** Chronic physical illness affects not only patients but also their partners. Dyadic coping (DC)—the ways couples cope in dealing with a stressor such as chronic illness—has received increased attention over the last three decades. The aim of the current study was to summarize the state of research on DC in couples with chronic physical illnesses.

**Methods:** We conducted a systematic review of qualitative, quantitative, and mixed-methods studies published between 1990 and 2020, assessing DC in couples affected by severe physical illnesses. We used DC and related search terms for the literature search in *Psycinfo, Psyndex*, and *Medline*. Five thousand three hundred thirty studies were identified in three electronic databases and 49 of these were included in the review (5,440 individuals reported on 2,820 dyads). We excluded studies on cancer, cardiovascular disease, and multiple sclerosis because of existing reviews in the respective fields. Half of the studies included were on diabetes. Other studies were on arthritis, chronic obstructive pulmonary disease (COPD), cystic fibrosis, human immunodeficiency virus (HIV), Huntington's disease, lupus erythematosus, Parkinson's disease, renal diseases, stroke, and endometriosis. Two raters extracted data using a predefined protocol, including study quality. Results were collated in a narrative synthesis organized by illness and DC operationalization.

**Results:** Overall, DC was associated with beneficial outcomes in physical health, well-being, and relationship satisfaction. Differential effects became apparent for certain chronic conditions potentially depending on certain disease characteristics, such as early-onset, sudden-onset, or life-threatening conditions.

**Conclusion:** Facing challenges together as a couple seemed indispensable for adapting to a diverse range of demands related to chronic illnesses with some specific demands of particular chronic diseases. There is a need for the development of truly dyadic interventions with an eye on the specific challenges of the various chronic conditions.

## Introduction

The emergence of chronic diseases is currently seen as the predominant challenge to global health (Bauer et al., 2014). Macrosocial and macroeconomic forces are understood as major determinants of population increases in chronic disease (Stuckler, 2008) with around half (50.9%) of adults in the US having at least one chronic condition (Ward and Schiller, 2013). Chronic diseases may deteriorate, enter remission, or fluctuate, but their defining characteristic is persistence (Helgeson and Zajdel, 2017).

To control or inhibit disease progression and to minimize disease side effects and disruptions to daily living, patients need to cope with the chronic condition. On the one hand, this entails behaviors such as taking medication, monitoring diet, exercising, and following up with health care professionals (Helgeson and Zajdel, 2017). Beyond these general tasks, some disease-specific tasks pose particular challenges and stressors, for instance adherence to time-consuming cancer treatment with severe side effects and uncertain outcomes, while in diabetes it is more about monitoring blood sugar and living a balanced life. On the other hand, patients need to deal with limitations in their daily lives, aspects of social and work life and adjustments to previously anticipated life plans. Whereas, disease refers to the undesirable biological processes that affect individuals, illness refers to the person's experience of the disease, including its psychological and social effects (Charmaz and Rosenfeld, 2010).

Historically, the majority of research on coping with physical illnesses focused on patients' individual processes. However, it is increasingly recognized that chronic illness takes place in an interpersonal context. The illness affects not only the person but also that person's spouse or romantic partner, family, and social network (Helgeson and Zajdel, 2017).

How couples cope with stress together has been the focus of research on dyadic coping since the early 1990s (DeLongis and O'Brien, 1990; Coyne and Smith, 1991; Revenson, 1994; Bodenmann, 1995). Dyadic coping (DC) refers to how partners communicate about stress, support each other in times of stress and deal with stressors together (Bodenmann, 1997). Dyadic coping represents a dynamic transactional process in which one partner's coping is dependent on the other's. Stressors could be either daily hassles or major stressors like chronic illnesses. Four facets of dyadic coping (DC) are distinguished (Bodenmann, 2005): common dyadic coping, when both partners are directly affected by the stress and work together to deal with it as a unit; supportive dyadic coping, in which one person is primarily affected and the other assists in their stress management; delegated dyadic coping, in which one partner is affected and the other takes over several tasks to reduce stress; and negative dyadic coping, where the partner attempts to help the actor cope but does so ineffectively, without motivation or in an ambivalent, superficial or hostile way. Apart from the Systemic Transactional Model (STM; Bodenmann, 2005; Bodenmann et al., 2016), communal coping (Lyons et al., 1998), marital or couple coping (Revenson, 1994; Revenson et al., 2005b), collaborative coping (Berg and Upchurch, 2007), or relationship-focused coping (DeLongis and O'Brien, 1990; Coyne and Smith, 1991) suggest theoretical frameworks for understanding coping processes in intimate relationships. For an integrative overview of DC models, see Falconier and Kuhn (2019) and for theoretical frameworks on couples' coping with chronic illness, see Revenson and DeLongis (2011). In the context of chronic illness, DC may be understood along a continuum of partner involvement from non-involvement (the patient perceives that they are coping individually), joint problem-solving, and shared emotion regulation (Revenson and Hagedoorn, 2019) to overinvolvement of the spouse (e.g., the patient perceives the spouse as controlling, engaging in miscarried helping) (Berg and Upchurch, 2007). As DC approaches are rooted in stress and coping literature (e.g., Lazarus and Folkman, 1984), it has long been a field that has developed independently from the social support literature and particularly emphasizes the stress-coping interplay between partners dealing with adversity and illness. It is only recently that both fields have begun to merge (Cutrona et al., 2018) and partner support theories have started to focus on the dyadic and dynamic nature of support as well (Donato et al., 2020). For theoretical clarity, in this review we focus exclusively on DC studies, as only DC approaches address common, joint or collaborative DC, which proves to be particularly relevant in illness management (Revenson and DeLongis, 2011).

Even though DC research emphasizes couple dynamics, the patient has quite often been the focal point in reporting on the couple's DC, thereby limiting information on the genuine dyadic nature of coping. However, recent studies mostly address patient and partner perspectives and their involvement in DC, yielding a more comprehensive understanding of DC in chronic illness (Badr and Acitelli, 2017).

The literature on DC and physical illness includes a wide array of chronic illness conditions. However, the standard of knowledge in terms of physical illnesses covered in DC literature and specific differential findings for the various chronic conditions is currently unclear. Overall, there is a consensus that psychosocial adjustment of the patient is enhanced when patients perceive their partner to be involved in dealing with the illness, but only to a certain degree. Some forms of DC (such as overprotection, controlling, or protective buffering) are perceived as detrimental to adjustment (Hagedoorn et al., 2000; Berg and Upchurch, 2007; Langer et al., 2009). Furthermore, as demonstrated in a cardiac population, partner hostility is negatively associated with patient engagement with the treatment (Rapelli et al., 2020). Additionally, for cardiac patients, not only high levels of negative DC but also low levels of positive DC were detrimental to the patient (Rapelli et al., 2021). Nevertheless, there is a need for a thorough overview of chronic illnesses that vary in terms of their timeline, consequences, level of control, and effects on identity coupled with in-depth consideration of the temporal process within DC in the context of chronic illness (Berg and Upchurch, 2007).

Thus the present narrative review sought to synthesize research on DC in couples affected by severe chronic physical illnesses. We included qualitative, quantitative, and mixed-methods studies in order to provide a comprehensive, complete picture. The research questions were the following: (1) How was the DC construct operationalized in the studies on chronic physical illness (dependent or independent variable or mediator/moderator) and what outcomes was it associated with? (2) How does the relevance of DC vary between different types of chronic physical illnesses depending on the extent of the supposed impact the illness has on relationship functioning?

## Methods

A systematic review of DC in couples suffering from chronic physical illnesses was undertaken as part of a larger global systematic review of DC. Methods of the analysis and inclusion criteria were specified in advance and documented in a protocol. Where applicable for the narrative review, we followed the PRISMA guidelines (Liberati et al., 2009).

### Literature Search

As part of a more general systematic review, we conducted a literature search on May 20, 2020 of the following electronic databases in the field of psychology: *Psycinfo, Psyndex*, and *Medline*. Search terms regarding DC were “dyadic coping,” “communal coping,” “couple coping,” “collaborative coping,” or “relationship-focused coping” published between 1990 and 2020.

Within identified sources, for this review we extracted only those references that focused on chronic physical illnesses. We used the following study and report eligibility criteria for selecting studies: (a) a focus on DC in romantic couples (or related terms such as collaborative coping or relationship-focused coping), (b) one partner suffering from a specific chronic illness (samples with mixed diagnoses were excluded to allow for contrast and comparison of specific chronic conditions), (c) articles published after 1990, since the first publications on the notion of dyadic coping emerged in the early 1990s (among others Bodenmann and Perrez, 1991; Coyne and Smith, 1991; Revenson, 1994; Lyons et al., 1995), (d) reporting on empirical data, (e) published in English, German, Spanish, French, or Portuguese since these are the languages with the most research activity in DC research, (f) published as a peer-reviewed article, book, editor-reviewed book chapter, or dissertation. When studies were published in parallel in a dissertation and peer-reviewed journal articles, we drew on the journal articles.

We excluded studies that either dealt exclusively with individual processes or outcomes or reported on social support, spousal support, or dyadic adjustment only. This ensured that only studies were considered that focus on dyadic appraisals, support responsiveness, and DC in dealing with physical illness as a stressor in order to warrant at least a certain level of homogeneity. Including the literature of the associated concept of spousal support was beyond the scope of this review.

### Study Selection

**Figure 1:**
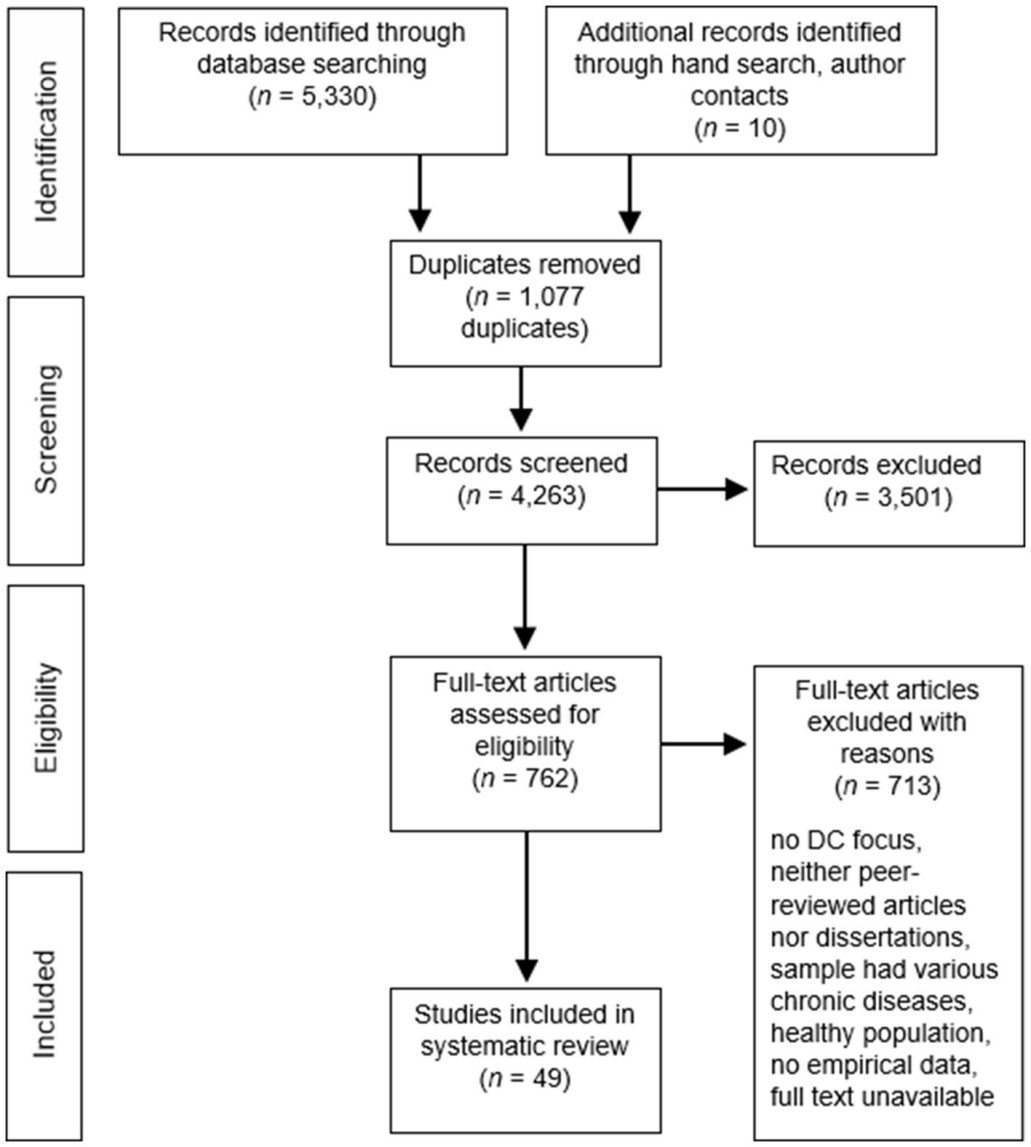
Flow chart.

The initial search yielded 5,330 publications. Figure 1 shows the flow chart of the study identification, retrieval, and the number of eligible articles. A senior researcher and two PhD students, assisted by graduate students, carried out screenings and data extraction. Studies were double-checked and where there was disagreement coders consulted with other members of the coding team until agreement was reached. We removed 1,077 duplicates and screened the titles and abstracts of the remaining references for eligibility. Three thousand five hundred one publications were excluded at this step. Where there was insufficient information, references were carried to the next step, in which full texts were retrieved and screened. When full texts were not available, authors were contacted if we could find an address. Unfortunately, quite a number of unavailable sources were dissertations. Ten references were added after hand searches of reference lists of included articles or thanks to authors who provided us with their full-text publications. After assessing the full texts, 713 additional sources were excluded.

While sorting the included publications, we came across existing current reviews on certain physical illnesses. Where this was the case, we referred to the reviews and excluded these illnesses from the current review. This included the following physical illnesses: cancer (McLean and Jones, 2007; Baik and Adams, 2011; Badr and Krebs, 2013; Li and Loke, 2014a,b; Traa et al., 2015; Kayser et al., 2018; Luo et al., 2020), cardiovascular disease (Trump and Mendenhall, 2017), chronic pain (Prenevost and Reme, 2017), and multiple sclerosis (Busch et al., 2014). To avoid double counting of samples, we juxtaposed authors' names, study designs, sample sizes, and outcomes of included publications. The final sample of studies included 49 publications based on 33 distinct data sets.

### Data Extraction and Quality Appraisal

We followed the principles of narrative synthesis to explore relationships within and between qualitative, quantitative, and mixed methods studies (Popay et al., 2006; Hong et al., 2017) and developed a data extraction sheet. Two researchers extracted data from each paper independently using a predefined protocol, resolving disagreements by discussion. We entered each publication into a table (see Supplementary Material) sorted by study type (qualitative, quantitative, intervention trial, or mixed methods). In these tables, we identified the authors, title, year of publication, study design, sample (age, gender, relationship duration, country, illness), DC measures, non-DC measures, type of intervention (where applicable), measurement time points, and main findings.

In order to evaluate study quality, we included a quality assessment for all publications. We used different assessment tools for intervention studies, quantitative, and qualitative studies. Mixed methods studies were assessed with both the qualitative and quantitative assessment tools. All questions were rated “yes,” “no,” or “cannot determine.” For intervention studies, we used the 14-item Study Quality Assessment Tools for controlled intervention studies of the National Institute of Health (NIH; National Institute of Health, 2014a). Study quality was rated as “good” when assessors answered “yes” to eleven or more questions, “adequate” in the case of six to ten answers of “yes,” or “poor” in the case of less than six answers of “yes.” For quantitative studies, we utilized an adapted version of the NIH Quality Assessment Tool for Observational Cohort and Cross-Sectional Studies (National Institute of Health, 2014b). We excluded five items that referred to cohort studies only, leaving nine questions. Study quality was rated as “good” when assessors answered “yes” to seven or more questions, “adequate” in the case of five or six answers of “yes,” or “poor” in the case of less than four answers of “yes.” Quality assessment of qualitative studies was based on the 10-item checklist of the Critical Appraisal Skills Programme (CASP; Critical Appraisal Skills Programme, 2018). As proposed by Lehane et al. (2017), studies received a quality rating of “good” when assessors answered “yes” to eight or more questions, a rating of “adequate” for six or seven answers of “yes” and a rating of “poor” for less than six answers of “yes.” We refrained from excluding studies that had low quality assessment ratings, since quality appraisal was only a rough estimate of study quality and the concordance and comparability of quality ratings between the assessment tools could not be verified. Most included publications were deemed “adequate” (*k* = 33, 67.4%), with some rated “good” (*k* = 12, 24.5%) and two (4.1%) rated “adequate” to “good,” while only two publications (4.1%) were lacking in rigor and thus rated “poor.”

### Data Analysis

We deemed a narrative synthesis approach an appropriate method to synthesize our data, since this allows the inclusion of qualitative, quantitative, and mixed methods studies. It adopts a textual approach allowing interpretative synthetization and evaluation of the findings. In our adapted narrative synthesis, we followed three stages of the narrative synthesis (Popay et al., 2006): (1) developing a preliminary synthesis; (2) exploring relationships within and between studies; and (3) assessing the robustness of the synthesis. The preliminary synthesis involved writing a short descriptive summary of each study. To ensure consistency, these narrative descriptions were produced in a systematic way; the same set of information was extracted from each study (Popay et al., 2006). Narrative evidence was clustered according to the type of physical illness. Within each chronic physical illness, findings were written up with the aim of condensing similar and overlapping findings and at the same time keeping the complexity of differential findings. Additionally, we appraised the robustness of the studies through the quality assessment process. Less robust study findings were pointed out for individual studies only in cases of contradictory results or when deemed relevant. Mostly, this pertained to studies lacking in test power or failing to use valid and reliable measures. Overall evaluation of study quality will be addressed in the discussion section.

One way to cluster the various illnesses is to draw on specific aspects of the disease which are relevant for coping: (1) How do the symptom and treatment burden (Sav et al., 2015) impact on the couple's relationship, on their sexual relationship, and on work and social activities (Bouras et al., 1986)? (2) How much care and taking over of tasks is required of the healthy partner? (3) Additionally, we would expect to find the greatest impacts on the individual as well as the couple in life-limiting (timeline) and degenerative illnesses (level of control). Self-evidently, this effort to cluster chronic physical illnesses can only be a crude approximation: depending on the degree of severity or the stage of the illness or comorbid mental or physical health, the impact on the couple may vary greatly within and between diseases. Following these aforementioned facets of chronic illness, we propose three clusters based on theoretical reflections. It is assumed that life-limiting and degenerative illnesses such as cystic fibrosis, Parkinson's, COPD, and renal diseases have a particularly strong impact on a couple's relationship (cluster I). Other diseases are less life-limiting but still have a high impact on the couple, with the affected individual needing care support from the partner on a regular basis or episodically, as in the case of individuals with arthritis or stroke survivors, for instance. Additionally, diabetes mellitus which has a strong impact on the couple in terms of lifestyle and diet (cluster II). Other chronic diseases affect the marital relationship or the sexual relationship, in particular diseases such as human immunodeficiency virus (HIV) or endometriosis (cluster III).

## Results

Forty-nine publications based on thirty-three data sets were included in this review (*k* = 21 quantitative, *k* = 10 qualitative, and *k* = 2 mixed-methods samples; for details of extracted data see Supplementary Table 1: quantitative studies, Supplementary Table 2: qualitative studies, Supplementary Table 3: mixed methods studies, Supplementary Table 4: intervention studies). The studies differed with regard to the underlying DC model. Hence the operationalization of DC in the quantitative studies varied considerably, mainly using instruments created by the authors *ad hoc* for the specific purpose of the study (*k* = 9, 21.4%); 6 (14.3%) studies used the Dyadic Coping Inventory (Bodenmann, 2008), a further 6 (14.3%) publications used a behavioral coding of DC, Ways of Giving Support (Buunk et al., 1996) was utilized by 3 (7.1%) studies, 3 (7.1%) publications used the Overprotection Scale (Hagedoorn et al., 2000), another 3 (7.1%) studies measured DC with the proportion of first-person plural pronouns use, and 12 (28.6%) studies used other reliable DC measures (see Supplementary Table 1). With respect to DC operationalization in the study design, of the 25 quantitative studies, DC was the independent variable in 14 publications (56%), the dependent variable in four publications (16%), both dependent and independent in two publications (8%), independent and moderator in two publications (8%), mediator in one study (4%), and moderator also in one study (4%). One quantitative study did not fit into this categorization because it assessed a newly developed DC measure.

For longitudinal studies, study duration ranged from 14 days (Zajdel et al., 2018) to 36 months (Vaske et al., 2015). Sample size varied greatly. For quantitative studies, total sample size was *n* = 4,736 (*M* = 225.52, *SD* = 162.15) and ranged between 20 dyads (*n* = 40; Robinson-Smith et al., 2016) and 280 dyads (*n* = 560; Trief et al., 2016). For qualitative studies, total sample size was *n* = 446 (*M* = 40.55, *SD* = 21.38) and ranged between seven dyads (*n* = 14; Robinson-Smith and Mahoney, 1995) and 36 dyads (*n* = 72; Rispel et al., 2012). The mixed methods studies had a total sample of *n* = 298 participants (*M* = 149.00; *SD* = 85.00) and a range between 32 dyads (McCarthy, 2012) and 117 dyads (Gamarel, 2014; Gamarel et al., 2016).

Regarding type of physical illness, 23 out of 49 (46.9%) publications examined diabetes, 8 (16.3%) studies concerned arthritis, 4 (8.2%) studies examined HIV, 4 (8.2%) studies concerned stroke, 3 (6.1%) studies examined COPD, 2 (4.1%) studies examined Parkinson's disease, 2 (4.1%) studies focused on renal diseases, and single studies (2.0%) focused on cystic fibrosis, endometriosis, and lupus erythematosus.

Ethnic and cultural diversity was low. The majority of data sets were from the US (23 out of 33, 69.7%, with mostly Caucasian samples), six data sets were from Western Europe (18.2%), two data sets from Sub-Saharan Africa (6.1%), one was from Israel (3.0%), and one with a mixed sample was from the US and Australia (3.0%). Heterosexuality was the norm, with the exception of HIV-positive samples, where both homo- and heterosexual couples were included. We present the evidence organized by the three clusters of impact and sort results within each cluster by chronic condition. We focused on results regarding DC as an independent variable, dependent variable, or moderator/mediator, and structured the presentation of results accordingly for each chronic condition. Furthermore, the focus was on evidence from longitudinal data followed by intervention trials, cross-sectional, correlational data and psychometric studies. This quantitative evidence was then complemented by mixed methods and qualitative evidence where available.

### Cluster I

#### Chronic Obstructive Pulmonary Disease

Chronic obstructive pulmonary disease (COPD) is a progressive lung disease characterized by shortness of breath and coughing, often caused by tobacco smoking or indoor and outdoor air pollution (Pauwels et al., 2001). Shortness of breath may lead to fewer activities, thus affecting couples' shared activities and their social life. Additionally, the healthy partner may need to take over tasks. For COPD, three publications based on two quantitative data sets were included (Meier et al., 2011, 2012; Vaske et al., 2015). COPD patients in these samples were mostly male. DC was examined using correlational designs, but two publications each reported data on DC as the dependent variable and DC as the independent variable.

When DC was examined as the independent variable, in a German 3-year longitudinal study (Vaske et al., 2015), patients' quality of life at follow-up was positively influenced by partners' stress communication at baseline. Partners' quality of life at follow-up was negatively influenced by patients' negative DC and positively influenced by partners' delegated DC as rated by patients. The authors did not report any effect sizes on these analyses. The results of the study suggested that partners of COPD patients strongly supported the patients but received little support in return. Patient perceived imbalance in delegated DC was associated with lower quality of life in couples (Meier et al., 2011). Quality of life of partners seemed to benefit when patients were able to communicate about their stress and provided delegated DC on their part.

For the publications with DC as the dependent variable based on one data set, the relevance of anxious and depressive symptoms for DC were examined (Meier et al., 2011, 2012). COPD patients with additional depressive symptoms were supported more often and attributed deficits in dyadic coping primarily to themselves. Partners with additional depressive symptoms reported more negative coping on their part and on that of their partners. Additionally, DC was compared in healthy couples with COPD couples (Meier et al., 2012). Compared with the healthy control group, COPD patients and their partners stated that the patients received more DC and were less able to provide their partners with support. In addition, couples with COPD perceived higher levels of negative DC and reported less positive DC (Meier et al., 2012). The authors did not report any effect sizes for these analyses.

#### Cystic Fibrosis

Cystic fibrosis is an inherited, progressive, and life-limiting disorder. Main symptoms include periodic respiratory infection, coughing fits, shortness of breath, intestinal blockage, diarrhea, and fertility problems. Affected individuals need to follow a strict treatment routine to manage symptoms and enhance life expectancy (Naehrig et al., 2017). This condition has severe effects on the marital relationship, because of the degenerative and life-limiting nature and dealing with uncertainty and daily challenges. One qualitative study from Israel (Werner et al., 2020) dealt with couples coping with cystic fibrosis (CF). Due to the early onset and life-limiting course of CF, couples were on average younger than samples with other chronic diseases. In the interview study, two main ways of dealing with CF as a couple were observed: cooperation vs. tension. Findings pointed to three dyadic qualities that supported coping as a couple, and distinguished between the two patterns of DC: mutual empathy, division of roles, and open, direct, and congruent communication about the illness.

### Cluster II

#### Arthritis and Lupus Erythematosus

Nine publications were included (*k* = 2 qualitative, *k* = 7 quantitative). Patients were mostly women suffering from rheumatoid arthritis (RA) or osteoarthritis, or, in one study, osteoarthritis with comorbid diabetes and lupus erythematosus. Arthritis is a general term for various conditions that typically include symptoms of joint pain and stiffness. Arthritis is currently a leading cause of work disability in the US (Centers for Disease Control and Prevention, 2020). Arthritis can be a degenerative condition with high levels of comorbidity, affecting the couple relationship in terms of care and taking over of tasks by the non-affected partner while also impacting on the sexual relationship as well as work and social activities.

Improving DC for pain management was the focus of four publications based on two different interventions. One intervention comprised training of pain coping skills and couple skills designed to supplement and reinforce the patient's pain coping skills in couples in which one partner suffered from osteoarthritis with persistent knee pain. This intervention was tested in two different US-based RCTs (Keefe et al., 1996, 1999, 2004). The spouse-assisted cognitive behavioral coping skills training (SA-CST) emphasized active learning. Skills and communication behavior were practiced in group sessions and couples were encouraged to practice at home as well. Control conditions were arthritis education or individual coping skills training with or without additional exercise training. In both studies, intervention benefits were specific: spouse-assisted training, either alone or in combination with exercise training, was found to produce improvements in coping and self-efficacy, whereas exercise training, either alone or in combination with spouse-assisted coping skills training, produced improvements in physical fitness and muscle strength (Keefe et al., 1996, 2004). Additionally, patients undergoing SA-CST had significantly lower post-treatment levels of pain and psychological disability and showed less pain behavior post-treatment than patients in a control group receiving arthritis education combined with spousal support (Keefe et al., 1996, 1999). The beneficial effects of SA-CST on coping and self-efficacy were still visible at 12-month follow-up (Keefe et al., 1999). It is important to mention that this study was rated “poor” in our quality assessment process, mainly because the publication lacked data on the reporting of the sample, the method of randomization, and the treatment.

A Dutch non-randomized controlled trial tested the benefit of partner participation in cognitive-behavioral self-management group treatment for patients with RA (van Lankveld et al., 2004). The intervention with the partner attending did not differ from the intervention without the partner, either in terms of content or effectiveness post-intervention. At 6-month follow-up, those patients with the partner attending reported better communication. However, since the intervention was educational and covered the same content in both conditions, it may be deduced that specific content focusing on the couples' interaction might be called for.

DC was examined in a mediator analysis in one study from the US that focused on women with a current lupus flare-up and their husbands. Wives' perceptions of husbands' emotional responsiveness fully mediated the associations between husbands' support efforts and wives' marital satisfaction and depressive symptoms. In contrast, more problematic DC was interpreted as being less emotionally responsive, which in turn was associated with poorer well-being. For husbands, a similar picture emerged: husbands' feelings of emotional responsiveness fully mediated the associations between wives' emotional support and husbands' well-being, but only partially mediated the relationship between wives' problematic support and husbands' well-being (Fekete et al., 2007).

In a correlational study from the US, zooming in from the more global types of coping in couples, the focus was on dyadic effects in relation to pain management in RA (Dowdy, 1999). Being in a good marriage and synchrony between the amount of support the wife desired and received were significantly associated with reduced RA pain ratings (Dowdy, 1999).

One quantitative study published a new DC measure: In this study from the US, a new measure covering interpersonal efficacy in couples coping with rheumatoid arthritis was developed and tested. The measure comprised three subscales: arthritis problem solving and emotions, arthritis symptom management, and arthritis-related couple outcomes. It appeared to be a reliable instrument with parallel versions for patients and spouses (Sterba et al., 2007).

Different patterns of coping together could be seen in two qualitative studies (one from the UK and one from the US). Both studies identified three groups of couples' illness-related interactions and management of the illness of one partner and how couples tackle the illness together (Mann and Dieppe, 2006; Yorgason et al., 2010). In these two studies, two of the identified groups were very similar: a group that shared illness management and a group where the ill partner was seen in charge of disease management. In the UK study, the third group was characterized by conflict over illness management, whereas in the US sample the third group consisted of couples that reported a mix of individual and shared coping activities.

#### Diabetes Mellitus

Diabetes mellitus describes a group of metabolic disorders characterized by chronic hyperglycemia resulting from defects in insulin secretion, insulin action, or both. Over time, hyperglycemia leads to serious damage to many of the body's systems, especially the nerves and blood vessels (World Health Organization, 2021b). The effects of diabetes on a couple's relationship vary but are most commonly related to dietary changes and physical activity and may require a partner to give care. Twenty-three publications (*k* = 3 qualitative, *k* = 20 quantitative) based on ten independent samples were included in this review covering type 1 and type 2 diabetes. All samples were from the US, with the exception of one Dutch study.

DC as the dependent variable was investigated in one publication that was part of four based on one sample with couples facing type 2 diabetes (US Midwest sample, see below). Here, having more confidence in the viability of the intimate relationship and finding satisfaction in making sacrifices for one's partner were related to engaging in more conversations about the patient's illness, inquiring about how the patient is feeling, and adopting a communal orientation toward diabetes, for both men and women (Johnson et al., 2013b).

Most non-intervention studies focused on DC as the independent variable, some including additional moderating variables such as gender, race, and attachment style. In young adults with type 1 diabetes, partner overinvolvement in diabetes management had a mixed impact on outcomes, whereas partner underinvolvement was uniformly related to poor outcomes. Only a small number of young adult patients considered their illness to be a shared problem and were actually aware that their illness was a source of distress to their partners, thus making it hard to ask for support (Helgeson, 2017).

Three publications were based on a 14-day longitudinal diary study of middle-aged couples with one partner having type 1 diabetes (Helgeson et al., 2019; Berg et al., 2020; Lee et al., 2020). Here, greater shared illness appraisals were associated with fewer self-regulation failures and better self-care. Collaborative and support strategies were more detrimental for diabetes management when individuals viewed diabetes as a less shared phenomenon (Berg et al., 2020). In the same study, DC was highest when patients and partners were consistent in their shared appraisals. High DC was related to better psychological and physical health (Helgeson et al., 2019).

One study from the US contributed mixed results, some contrary to predictions: there was no evidence that collaboration was directly related to better disease management or emotional well-being for patients or spouses. Spouse collaboration did not significantly interact with dyadic appraisal to predict spouses' enjoyment of the relationship, but patient collaboration did. Disease-related collaboration was more beneficial for partners who viewed disease management as a shared responsibility (Hemphill, 2015).

Eight different publications were based on one US sample of couples with recently diagnosed type 2 diabetes couples (Helgeson, 2017; Van Vleet et al., 2018, 2019; Zajdel et al., 2018; Helgeson and Van Vleet, 2019; Van Vleet and Helgeson, 2019; Helgeson et al., 2020a,b). In spouses and patients affected by type 2 diabetes, more observed communal coping by partners was linked to better perceived diabetes problem-solving and fewer negative affects for females but not for males, as well as higher relationship satisfaction in both males and females (Van Vleet et al., 2018). Communal coping was significantly linked to greater perceived problem resolution and more positive perceptions of the discussion, which led to increases in self-efficacy and decreases in diabetes distress in patients, but only patient communal coping was linked to changes 6 months later (Van Vleet et al., 2019). Communal coping on a daily basis helped both patients and spouses adjust psychologically to the illness and enhance patient self-care behaviors (Zajdel et al., 2018). However, there may be a limit to how much communal coping was adaptive. Patients who reported greater overlap with their partners in coping with diabetes (meaning more engaged partners) also saw those partners as overprotective and controlling (Helgeson et al., 2017). Additionally, communal coping may not be equally beneficial for everyone. In particular, communal coping was less beneficial for the relationship quality in patients with avoidant attachment (Van Vleet and Helgeson, 2019). Partners' communal coping, however, was more beneficial for patients than spouses and for women than men. Furthermore, white patients and black spouses benefited more from their own communal coping than black patients and white spouses (Helgeson et al., 2020a). Patient communal coping behavior interacted with partners' communal coping, such that partners' communal coping was related to lower patient distress, higher patient self-efficacy, and higher patient medication adherence, but only when partners scored low on unmitigated communion (Helgeson et al., 2020b). Inclusion of other in self (IOS) was related to communal coping and relationship quality (Helgeson and Van Vleet, 2019).

In a Dutch sample with type 1 and type 2 diabetes, higher received active engagement and lower received protective buffering predicted relationship satisfaction, in both patients and partners. Active engagement moderated the negative association between protective buffering and relationship satisfaction (Schokker et al., 2010). Four publications from Johnson's lab were based on one sample of couples facing type 2 diabetes (US Midwest sample; Johnson et al., 2013a,b, 2015; Trump et al., 2018), two of these investigated DC as the independent variable. Common DC was associated with higher levels of diabetes efficacy for both patients and spouses which was associated, in turn, with better dietary and exercise adherence of the patient (Johnson et al., 2013a). However, consideration must be given to nuanced associations between the different ways spouses cope with illness to achieve better diabetes outcomes. Spousal overprotection was indirectly associated with worse dietary adherence in patients with type 2 diabetes only when spousal active engagement was low (Johnson et al., 2015).

In a mediation analysis based on the data of the abovementioned middle-aged diabetes type 1 sample, spouse we-talk was more important than patient we-talk because it signified that spouses were involved in helping with diabetes management. This association was mediated by providing emotional support and refraining from criticizing the patient. Patient we-talk, however, was unrelated to well-being (Lee et al., 2020).

DC as the moderator was the focus of one publication of the abovementioned US Midwest sample. Spouses' evaluation of dyadic coping attenuated the direct paths between spouses' depression symptoms and patients' adherence to dietary regimens. Additionally, spouses' evaluation of DC attenuated the direct paths between spouses' acute stress and patients' adherence to dietary regimens (Trump et al., 2018).

In two qualitative studies, based on US samples with type 2 diabetes, authors reported the following findings: couples who talked about diabetes appeared to have greater support from the spouse and fewer difficulties with dietary adherence (Beverly et al., 2008). Beverly and Wray (2010) used the terms collective support, collective motivation, and collective responsibility, which may influence couples' adoption and maintenance of regular exercise. Because women provided the majority of support to older family members and served as gatekeepers for their health, they seemed more experienced and better equipped when it came to providing effective support for spouses living with diabetes than males (Beverly and Wray, 2010). A particular aspect of diabetes management is the focus on dietary adherence. In older couples, traditional division of labor may be prevalent and have an impact on DC. Depending on who is suffering from diabetes in the relationship, dietary management was seen as a form of control (sensed by male patients) or lack of support (sensed by female patients) (Beverly et al., 2008).

A qualitative interview study indicated that a particular stressor in dealing with diabetes was acute hypoglycemia, where a spouse needed to act quickly while the patient was sometimes resistant to any support due to the concomitant mood swings and irritability. It seemed there was a fine line between ongoing reminders and nagging or finding the right level of respect for the patient's need for independence and autonomy. Partners' fear of negative consequences of poor diabetes management led to more negative DC interactions (Trief et al., 2003).

A couple-oriented diabetes intervention was developed and tested in the US and published as a pilot study and randomized controlled trial (Trief et al., 2011, 2016). The intervention was a telephonic intervention (“couple call,” CC, 12 sessions): In addition to diabetes management, couples were encouraged to provide mutual support for nutritional and behavior change, using collaborative problem-solving techniques and recognizing their interdependence. Control conditions were individual phone intervention (“individual call,” IC, 12 sessions) and individual diabetes education only (“diabetes education,” DE, 2 sessions). Intention-to-treat analyses found significant hemoglobin level (A1C) reductions for all groups with no differences between arms. Subgroup within-arm analyses found that the CC intervention was efficacious in lowering A1C levels for individuals with high A1C levels. For body mass index (BMI), CC showed significant improvement, and CC and DE led to decreased waist circumference. Unfortunately, Trief and colleagues (Trief et al., 2011, 2016) did not include any DC outcome measures and hence changes in DC following the intervention could not be captured.

#### Parkinson's Disease

Parkinson's disease is a neurological disorder that mainly affects the nervous system. Currently, there are no treatments that slow the neurodegenerative process and it is difficult to manage the symptoms (Kalia and Lang, 2015). Due to its progressive and complex nature, Parkinson's affects the couple relationship on many levels: the marital and sexual relationship, work and social activities as well as taking over household and care tasks by the partner as the condition progresses. DC in couples affected by Parkinson's was the focus of one quasi-experimental pilot intervention study and one qualitative study, both from the US.

The pilot study reported a quasi-experimental intervention study testing the efficacy of *Strive to Thrive*, a group workshop on self-management for Parkinson's disease. The intervention covered self-management skills related to monitoring, taking action, problem-solving, decision-making, and evaluating results. Significant changes were observed only in spouses' mental relaxation techniques but not for dyadic outcomes such as active engagement (Lyons et al., 2020). Due to the quasi-experimental design of the study with no randomization and a small sample size, study quality had to be rated as “poor” in our quality assessment.

In the qualitative interview study on the effect of Parkinson's on couples, even though participants were not asked directly about DC, they touched on relevant aspects of DC, such as emotional support, listening, providing informational support, giving advice, or encouraging shifts in perspective. However, participants also described difficulties in coping together: partners' differing approaches to coping impeded support or support threatened independent and capable identity or placed unwanted emphasis on the disease. Furthermore, there was a fear of burdening the other partner and actual draining of the caregiver, in addition to having to deal with dependency. DC was enhanced by framing partners as equals, relinquishing control, sharing humor, seeking/providing support indirectly or subtly, and taking the other's perspective (Martin, 2014).

#### Renal Disease

Chronic renal disease can have a number of causes and describes the gradual loss of kidney function (Parmar, 2002). Due to the often life-limiting and disabling nature of chronic renal diseases, the effects of these conditions on the couple are complex and may include care tasks by the healthy partner, restrictions on social and work life, and in the case of dialyses time-consuming treatment regimens. The two DC studies included in the review, one cross-sectional study from Germany and one qualitative study from the US, focused on the treatment of chronic renal diseases, namely dialysis and kidney transplantation.

The German-based cross-sectional study examined gender and role differences. Couples with male patients found that female caregivers showed higher own supportive DC than the males. In couples with female patients, women reported greater communication of their own stress, supportive DC, total positive DC, and total DC as well as depression compared to men. Low DC discrepancies were associated with positive psychological outcomes. In couples with female patients, higher comparability was associated with higher DC. Lower levels of similarity for male spouses showed correlations with higher rates of depression and anxiety in the females (Tkachenko et al., 2019).

The qualitative study assessed how renal patients and their partners experienced the short daily home hemodialysis and what kind of relationship factors were beneficial. Four profiles for dyadic coping emerged from the analysis of couple interviews: (1) thriving—patients' care partners flourished; (2) surviving—strong couples adjusted to challenges; (3) martyrdom—one partner defers their needs and resentments to make dialysis work; and (4) seeking another option—patients are unwilling to burden an anxious partner. Associated with the thriving dyads was dialysis training that was unhurried and valued care partners as well as patients used a mix of learning strategies and provided a home visit for the first home treatment (Wise et al., 2010).

#### Stroke

Stroke is a condition where blood flow to the brain is compromised, causing cell damage, either through a lack of blood flow (ischemic) or bleeding (hemorrhagic). Stroke is a major cause of disability and the second most common cause of death worldwide (Donnan et al., 2008). For those stroke survivors with disabling brain damages, a phase of regaining and relearning skills ensues, which can be stressful for the survivor as well as the partner. Depending on the level of disability, the marital relationship can be minimally or severely effected in different areas, for instance due to loss of language skills, care tasks taken over by the partner, and fear of future strokes. Dyadic coping in stroke survivors and their partners was the focus in four studies (*k* = 2 intervention studies, *k* = 1 mixed-methods study, and *k* =1 qualitative). All studies were conducted in the US.

The two pilot studies from the US tested the feasibility of two different post-stroke couple interventions. One pilot study looked at a self-administered dyadic positive psychology-based intervention for stroke survivors and their partners where either one or both reported depressive symptoms (Terrill et al., 2018). Feasibility was established informing a larger, planned randomized-controlled trial. In the second pilot study (Robinson-Smith et al., 2016), psychoeducational intervention was adapted from the *Partners in Coping Program* (Kayser and Scott, 2008) and tailored to couple's needs, focusing on present, reasonable goals, reframing, spousal communication, integrating body image, and incorporating social and pleasurable activities. First, yet underpowered, results indicated a significant increase in experimental group stroke survivors' independent coping and quality of life and a reduction of depressive symptoms. Significant increases in positive DC were observed in experimental group spouses.

In the mixed methods study, cross-sectional data showed that lower levels of active engagement and higher levels of protective buffering were associated with greater depression in spouses but not in stroke survivors. The qualitative arm of the mixed methods study revealed, amongst other things, accounts of relationship challenges as well as growth as a couple, with some couples talking about how the illness had brought them closer together (McCarthy, 2012). The qualitative study captured how couples sought a new equilibrium together after the stroke in an attempt to reduce its impact and adapt to changes in formerly shared leisure activities as well as division of homemaking and breadwinning labor (Robinson-Smith and Mahoney, 1995).

### Cluster III

#### Endometriosis

Endometriosis is a condition in which tissue similar to the uterine lining (endometrium) grows outside of the uterus. It is characterized by dysmenorrhea (painful menstrual periods), chronic pelvic pain, dyspareunia (pain during intercourse), and infertility (As-Sanie et al., 2019). For these reasons, endometriosis affects the couple relationship in terms of dealing with pain and family planning as well as the sexual relationship. A qualitative study with a US- and Australian-based sample of ten couples focused on dyspareunia due to endometriosis and its effects on the couple relationship (Brown, 2007). Most women in the study had developed endometriosis before entering the relationship, and thus they were openly sharing with their partners about their condition and had the impression that they were coping as a team. The women in this study seemed to be the ones receiving the information and sharing it with their partners. The women stressed how important it was to talk about the illness and their relationship. The partners talked about how over time they had come to understand their partners' cycles, moods, and triggers. Communication about the dyspareunia seemed particularly important for adapting their intimate relationship to avoid pain.

#### HIV

The human immunodeficiency virus (HIV) weakens the immune system. The most advanced stage of HIV infection is acquired immunodeficiency syndrome (AIDS), which is defined by the development of certain cancers, infections or other severe long-term clinical manifestations. To date, for those with access to effective HIV prevention, diagnosis, treatment and care, HIV infection has become a manageable chronic health condition (World Health Organization, 2021c). Depending on access to effective prevention and treatment, effects on the couple relationship may vary. The sexual relationship is affected in serodiscordant couples. For couples without effective treatments, which is more often the case in the global South, HIV/AIDS can be life-limiting, potentially for both partners, which represents a severe stressor.

Three qualitative and one quantitative study (from three samples) on HIV were included in the review. Two studies were from Sub-Saharan Africa with heterosexual serodiscordant couples and one from the US with male–male, mostly serodiscordant couples.

The publications from the US were based on data from the Duo Project (Conroy et al., 2016) studying homosexual couples in which at least one partner was HIV-positive. In a quantitative longitudinal study (Gamarel, 2014), DC was operationalized as the inclusion of the partner in self and DC communication patterns. Communication was examined as the dependent variable.

Both sets of partners' reports of higher positive communication scores were associated with increased relationship satisfaction, and higher negative communication scores were associated with decreased relationship satisfaction. Only the actor effects were significant in any of the actor–partner interdependence models (APIMs), meaning that one's own level of positive or negative communication was relevant, not the partner's level of communication. Additionally, higher positive communication and lower negative communication scores were associated with sexual satisfaction and lower levels of depressive symptoms. However, HIV-negative partners' negative communication scores were associated with a decrease in non-adherence and a decrease in anal sex. Additionally, a mediation effect was analyzed, suggesting that partners who reported higher levels of inclusion of the partner in the self at baseline also tended to report higher levels of positive communication at 6-month follow-up and, in turn, higher levels of relationship satisfaction at 12-month follow-up (Gamarel, 2014).

In the qualitative study arm (Gamarel et al., 2016), participating couples (*n* = 20) were grouped into two basic orientations toward health, the “relational” and the “personal.” The relational orientation described health as interconnected and couples prioritized being aware of one another's health status and care needs. They emphasized open communication and empathetic concern independent of serodiscordant status. A subgroup was termed “asymmetrical,” denoting couples in which one partner's health was prioritized over the other's. The personal orientation group consisted of couples in which one or both partners described their health and health care as independent and autonomous (as long as health status was stable). Individuals with more autonomous orientations to their own and their partners' health also were more satisfied with their partners' support.

In another qualitative study embedded in a larger RCT of vaginal gel use for the prevention of vaginally acquired HIV infection, couples were interviewed in Uganda and Zambia on their coping with HIV and serodiscordance (Montgomery et al., 2012). In Uganda, participating couples believed that joint effort was needed regarding the mutual acceptance of their serodiscordant status. They communicated about the situation and engaged in cooperative action to solve problems, including use of condoms and gel. However, in the Zambian sample, protecting the health of the HIV-negative woman was left to the women themselves. Communication with the spouse occurred only when the women sought permission to use the gel with the implicit threat of conflict or even partner violence if they did not seek this assent and used the gel secretly. These Zambian couples did not touch upon any facets of DC in their interviews, thus depicting the absence of positive DC.

Finally, a qualitative study in South Africa and Tanzania focused on communication about HIV serodiscordance and dealing with HIV (Rispel et al., 2012). Half of the couples showed a coping style termed “sero-normalcy,” whereby couples saw themselves as “normal” even though HIV still seemed to affect the relationship. Another coping style was termed “sero-silent,” partners reporting that they did not talk much with each other about issues related to their serodiscordant status. The third coping style was termed “sero-sharing”: couples portrayed HIV as being an issue that they dealt with together, talking about health issues and their desire for parenthood. Some couples exhibited features of more than one coping style and, at times, partners differed in their ways of coping within a couple.

## Discussion

Chronic physical illness affects not only the patient but also the partner (Acitelli and Badr, 2005; Yorgason and Choi, 2016; Revenson and Hagedoorn, 2019). Over the last 30 years, the field of DC has emerged with the understanding that partners communicate about stress, support each other, and deal jointly with chronic illness. The present study aimed to synthesize research on DC in couples affected by severe physical illnesses. We included forty-nine qualitative, quantitative, and mixed methods studies. When looking at the overall findings, both partners dealt with chronic illness better, if (a) they engaged in stress communication, (b) their supportive DC matched the needs of the partner and (c) they shared similar illness perceptions. The findings of this review mirror the results of previous reviews on chronic diseases such as cancer (Traa et al., 2015), cardiovascular disease (Trump and Mendenhall, 2017), chronic pain (Prenevost and Reme, 2017), and multiple sclerosis (Busch et al., 2014).

Interestingly, the inclusion of qualitative and mixed methods studies may be viewed as a proof of concept regarding the facets of DC and its relevance for patients and partners alike when dealing with chronic illness. When couples were asked, as part of the qualitative interviews, to talk freely about their experience of dealing with physical illness, they regarded the relevance of stress communication, feeling supported in the right way by one's partner, and collaboration as positive across all qualitative studies. Similarly, they regarded the lack of DC as stressful. However, there was one exception regarding the beneficial stance of DC. Some couples in a study on male–male couples with HIV valued independence during phases of relatively good health whilst knowing that this situation was fluid and depended on the affected partner's health status (Gamarel et al., 2016). Furthermore, the inclusion of qualitative and mixed-methods studies revealed the relevance of further facets of beneficial DC, for instance adopting the other's perspective and using humor. Additionally, qualitative analyses often grouped together different phenomena of DC. Some couples seemed to deal well with the physical illness together, whereas others would benefit more strongly from outside support or DC interventions.

In the quantitative studies, DC was mostly operationalized as the independent variable, fitting theoretical models, mainly STM (Bodenmann, 2005) or relationship-focused DC (Coyne and Smith, 1991). However, especially this approach would be ideally suited to delving more into the dyadic coping processes in chronic disease by emphasizing observational studies that were extremely rare. Looking at how couples deal with the chronic illness in real time interactions might be highly beneficial and shed more light on the mechanisms of DC, the frequency and quality of partner's contributions, their impact on the patient, and consequences on quality of life and illness development.

In a first attempt to cluster the impact of chronic illness on couples, we collated type of illness in groups according to the assumed level of impact of symptom and treatment burden on the couple relationship. As mentioned above, this effort can only be a crude approximation. The impact may vary considerably, depending on the degree of severity or the stage of the illness or comorbid mental or physical health conditions of the affected individual as well as the partner. Based on the included studies and the variety of illnesses covered, at this juncture no clear picture emerged that would sufficiently differentiate DC findings between clusters. However, communication about the illness and the required support seemed highly relevant across the clusters. Secondly, in cluster II, it became apparent that overinvolvement could threaten the patient's independent and capable identity (cf. Table 1). Thus, the assumption that overinvolvement is deleterious (O'Brien and DeLongis, 1997) was only found in cluster II couples.

**Table 1:** Main DC findings for the various physical illnesses.

**Cluster**	**Physical illness**	**Main findings**	**No. of publications**
Cluster I	COPD	- Compared to a control group, (1) COPD patients received more DC and were less able to provide support to their partners, (2) couples with COPD perceived higher levels of negative DC and reported less positive DC. - Partners of COPD patients strongly supported the patients, but got back little support themselves. Partners' quality of life seemed to benefit when patients were able to communicate about their stress and provided delegated DC on their part.	3
	Cystic fibrosis	- Due to the early onset and life-limiting course of CF, couples were on average younger than samples with other chronic diseases. - Two main ways of dealing with CF as a couple were observed: cooperation vs. tension. Findings pointed to three dyadic qualities that supported coping as a couple, and distinguished between the two patterns of DC: mutual empathy, division of roles, and open, direct, and congruent communication about the illness.	1
Cluster II	Arthritis and lupus erythematosus	- Various types of DC patterns in affected couples: “shared illness management” and “ill partner in charge,” and “conflict over management” - Being in a good marriage and synchrony between the amount of support the female rheumatoid arthritis (RA) patient desired from her partner and received were significantly associated with reduced RA pain ratings. - Intervention: cognitive-behavioral self-management group treatment for patients with RA led to better communication at 6-month follow-up. - Intervention: training in pain coping skills and training in couples skills designed to supplement and reinforce the patient's pain coping skills led to improvements in coping and self-efficacy at 12-month follow-up. - Measurement instrument: A measure covering interpersonal efficacy in couples coping with rheumatoid arthritis was developed and tested.	9
	Diabetes mellitus	Type I - Mostly younger patients who lived with their chronic condition before entering into the couple relationship. Patients with type I diabetes considered their illness to be their individual problem and were often not aware that their illness was a source of distress to their partners, which made it hard to ask for support. - Spouses' we-talk might be more important than patient we-talk because it signifies that spouses are involved in helping with diabetes management, namely by providing emotional support and refraining from criticizing the patient. - For young adults with type 1 diabetes, partner overinvolvement in diabetes management had a mixed impact on outcomes, whereas partner underinvolvement was uniformly related to poor outcomes. - When patients and partners were consistent in their shared appraisals, DC was highest. High DC was related to better psychological and physical health. Type II - DC, in particular partner communal coping on a daily basis, was associated with higher levels of diabetes problem-solving, diabetes efficacy, and relationship satisfaction for both patients and spouses. - There may be a limit to how much communal coping is adaptive. Patients who reported greater overlap with their partners in coping with diabetes or patients with avoidant attachment style also saw those partners as overprotective and controlling. - Diabetes efficacy was associated with better dietary and exercise adherence on the part of the patient. - Differential effects were also observed for gender and ethnicity. - In older couples, traditional division of labor may be prevalent and have an impact on DC. Depending on who is suffering from diabetes in the relationship, dietary management is a form of control (sensed by male patients) or lack of support (sensed by female patients). - A particular stressor was dealing with hypoglycemia where a spouse might need to act quickly while the patient might be pushed into resistance to any support. Finding the right balance of respect for the patient's need of independence and autonomy was important.	23
	Parkinson's disease	- Beneficial aspects of DC were emotional support, listening, providing informational support, giving advice, or encouraging shifts in perspective. - Partners' differing approaches to coping can make support difficult or support may threaten independent and capable identity or place unwanted emphasis on the disease. - There was a fear of burdening one's partner and draining the caregiver and having to deal with dependency.	2
	Renal disease	- Complex sex and role differences occurred in terms of DC in renal transplant patient caregiver dyads. - Dyads successfully coping with home dialyses; unhurried training that valued care partners as well as patients, used a mix of learning strategies, and provided a home visit for the first home treatment was beneficial.	2
	Stroke	- Lower levels of active engagement and higher levels of protective buffering were associated with greater depression in spouses but not in stroke survivors. - Couples sought a new equilibrium together after the stroke in an attempt to resolve the impact of the stroke and adapt to changes in formerly shared leisure activities as well as division of homemaking and breadwinning labor.	4
Cluster III	Endometriosis	- The women seemed to be the ones obtaining the information and sharing it with their partners. Communication about the illness, the relationship, and the dyspareunia seemed particularly important.	1
	HIV	Heterosexual couples - To protect the healthy partner in a serodiscordant heterosexual relationship, joint effort was needed regarding the mutual acceptance of their serodiscordant status. Communication was important about the situation and engaging in cooperative action to solve problems, including use of condoms and gel. Homosexual couples - In homosexual couples, both partners' reports of higher positive communication scores were associated with increased relationship satisfaction. This was mediated by higher levels of inclusion of the partner in the self. - Couples with a “relational” orientation described health as interconnected and couples prioritized being aware of one another's health status and care needs. Couples with a “personal” orientation consisted of couples in which one or both partners described their health and health care as independent and autonomous as long as health status was stable.	4

In the following, we will focus on a number of further differential aspects that were apparent above and beyond the beneficial aspects of DC for individual chronic physical health conditions (see Table 1 for an overview). Firstly, a differential effect of DC in early- and late-onset illnesses became apparent across the included studies, which has already been discussed in a previous review (Berg and Upchurch, 2007). For instance, young people with type 1 diabetes who have had diabetes for many years and were diagnosed in childhood represent an interesting group. They have already learned to manage diabetes on their own before entering into a committed relationship and therefore DC might play another role compared to couples confronted with an emerging disease within their relationship (Berg et al., 2020). Dealing with their health condition independently may have been a relevant part of their individuation process while they were growing up. For the early-onset illness patients it seemed to be more difficult to share illness appraisals with their partners, as they perceive an imbalance of illness-related knowledge and skills. This experience seemed similar for couples with COPD and cystic fibrosis. On the other hand, in couples with late-onset illnesses, an understanding of the illness as a “we-disease” (Kayser et al., 2007) may be more logical, as the disease occurred during the intimate relationship. This shared experience of the onset might facilitate shared appraisals and mutual acceptation of the chronic illness as a shared task. Yet another picture emerged for couples in which the woman was suffering from dyspareunia due to endometriosis.

Secondly, another differential effect was visible in the case of stroke. A sudden onset out of the blue with related severe physical restrictions seemed to be particularly stressful to stroke survivors as well as their partners, with a high incidence of comorbid depressive symptoms in both (Robinson-Smith et al., 2016; Terrill et al., 2018). In these couples and in couples with Parkinson's disease, the fear of burdening the healthy partner and draining the caregiver were particularly visible.

Third, an additional facet of DC was captured in the case of serodiscordant couples with HIV/AIDS: not only caring for the ill partner but also preventing infection of the healthy spouse added a more vital note to DC. This was particularly visible in the Ugandan and Zambian sample of heterosexual couples and the threat to the woman's life if the partner was not concerned about her health (Montgomery et al., 2012). This aspect of DC was correspondingly visible in the US samples with HIV, however to a slightly less vital degree, since theses couples reported receiving HIV treatment leading to a suppressed viral load and pre-exposure prophylaxis for the non-HIV partner. Still, the relevance of open communication, trust, and collaborative coping in medication adherence have a high relevance for both partners not just the one with the illness.

Additionally, gender, cohort, and sociocultural effects were found across the included studies. Disentangling the role of patient/partner and the influence of gender is difficult (Revenson et al., 2005a). In light of traditional gender roles, women assume a disproportionate share of responsibilities for maintaining the family's organization and providing nurturance to family members, going along with higher expectations that they will provide DC (Revenson et al., 2005a). Adaptations in role responsibilities outside traditional gender roles lead to differential effects of disease, for instance in the case of diabetes, RA, or stroke. In a similar vein, Revenson et al. (2005a) described that due to the traditionally unequal division of household labor, a greater work load was placed on healthy female spouses when the male partner suffered from a chronic condition vs. getting outside help when the woman was the one with the chronic illness. Similar findings were reported in a systematic review on cardiovascular disease, with women in the role of DC providers reporting more distress (Trump and Mendenhall, 2017).

Sociocultural factors influence the expectations and norms governing the level of interdependence in committed relationships, with collectivistic cultures and females more likely to represent the self in relation to others (Triandis, 2001). Most studies, however, were conducted in the US or in Europe. We do not yet have a clear understanding of how couples from other parts of the world with a more collectivistic background deal with chronic illnesses together and how gender norms and culture interact with DC (see also Falconier et al., 2015). Again, the two included HIV studies seemed to yield particular findings, as they were conducted in Africa (Montgomery et al., 2012; Rispel et al., 2012). However, their contribution to the disentanglement of culture, gender, and illness is marginal, as HIV represents a specific challenge for DC, as this was the only contagious illness included in this review. Therefore, DC with HIV is not directly comparable with the other chronic illnesses affecting primarily only one partner and secondarily the other (in terms of worrying, care giving, inequity of roles, etc.). In sum, this review illustrates the need for more studies from different geographical areas and cultures all over the globe for a deeper understanding of the role of DC in couples with chronic illness.

It is important to keep in mind that DC does not happen in absence of other factors influencing the adjustment process when facing chronic disease. Particularly in older couples, people with chronic disease frequently face multiple chronic physical or mental health conditions (Vos et al., 2015; Helgeson and Zajdel, 2017). However, this pile-up of stress due to chronic disease, multiple other impairments, and regular daily demands has not been studied in the included publications. This would be important, however, for gaining a deeper insight into DC processes embedded in different age contexts, stages of the illness, the exhaustion of DC resources over time, or comorbidity (only one partner affected by a chronic illness vs. both).

A number of interventions have been developed to alleviate the burden of chronic physical conditions. Mostly, these interventions developed out of existing interventions with an individual focus that included the partner as a participant with varying degrees of adaptation to a partner perspective. Effect sizes of health and relationship benefits were in the smaller range and could potentially be increased with a more stringent focus on couple interaction, as is the case for example in Couples Coping Enhancement Training (Bodenmann and Shantinath, 2004).

Chronic diseases with high prevalence rates (>10 per 100 in at least one country) in the Western world are diseases of the nervous system, hypertension, headache/migraine, chronic respiratory disease, genitourinary diseases, osteoarthrosis, back and spinal cord disorders, skin diseases, and allergies (Dalstra et al., 2005). Disproportionately, in our review, the majority of studies were on diabetes. Interestingly, no studies were found on headache/migraine, genitourinary diseases, back and spinal cord disorders, skin diseases and allergies, or obstructive sleep apnea syndrome. Even though they may vary in severity and some chronic illnesses such as allergies require less DC, for other chronic physical illnesses, the lack of couple research is unfortunate, for instance regarding headache/migraine, skin conditions or back and spinal cord disorders, which are very common and burdensome for couples, affecting daily functioning as well as couples' sex lives. Another caveat concerns the fact that most studies were conducted in the US or Europe in high-income nations, while chronic disease is most widespread in low and middle income countries (World Health Organization, 2013, 2021a).

A criticism voiced by Berg and Upchurch (2007) is that the majority of the DC literature did not include physical health outcomes but rather relied heavily on mental health outcomes. The current review shows that over a decade later, the situation has changed. A great number of studies on various health conditions including physical health outcomes is available: diabetes (glycemic control), COPD (lung function), HIV (viral load), arthritis (aerobic fitness and strength). Outcomes pointed toward a general positive impact of DC on physical health. Another outcome that is quite often overlooked is the economic costs of chronic disease (even implicit ones), e.g., staying home from work for check-ups. Future research could analyze the potential affect DC has on economic outcomes for the couple too.

Importantly, most studies were cross-sectional. Prospective studies were rare. In future research, efforts should be made to collect longitudinal data, given the dynamic, transactional, and circular nature of DC. It is important to keep in mind that DC is not a static concept, but changes according to the nature of the illness (i.e., severity, restrictions, comorbidity, impact on the couple) and the couples' adjustment and change of preferences (Helgeson, 1993). Many factors not only affect DC but also are affected by it in turn. For instance, relationship satisfaction can increase the likelihood of DC and can be further enhanced by DC processes (Falconier et al., 2015).

In the current review of studies, the quality of quantitative studies was sometimes negatively affected by *ad hoc* formulation of items or the truncation of existing measures compromising psychometric properties. Additionally, most quantitative studies were lacking in power analysis and relied on non-representative convenience sampling. This problem is characteristic of different systematic reviews on chronic illness, e.g., on DC with cardiovascular disease (Trump and Mendenhall, 2017). Qualitative studies, on the other hand, often lacked information on how the researchers critically examined their own role or dealt with potential bias and subjective influence. Whether these shortcomings were due to journal space restrictions or unintentional remains unclear. One needs to bear in mind that reporting quality may differ from study quality (Soares, 2004). Another problem is related to multiple publications based on the same data sets. This practice might inflate interpretation of findings and overestimate their validity.

This review provides only a snapshot of a rapidly evolving research area. The studies identified included, for instance, some study protocols for intervention studies that seem to be currently underway (Wittmann et al., 2017; Lüscher et al., 2019). Future reviews should also overcome the distinction between DC and spousal support literature. Even though both fields developed in parallel, it is high time a metatheory linking the different approaches were formulated, as has already been attempted by Cutrona et al. (2018). The synthesis of knowledge from both fields would further stimulate the development of interventions for couples facing chronic physical illness.

### Strengths and Limitations

A number of limitations pertaining to our review need to be taken into account when one considers our findings in addition to the above limitations of the included studies. Firstly, a strength of our study was the additional inclusion of gray literature (e.g., unpublished dissertations), often excluded from systematic reviews even though they may provide thorough insights. Despite great efforts, we could not obtain several dissertations either because we were unable to contact the authors or because the authors failed to respond. Hence in some areas the body of knowledge may be incomplete in this review.

In some cases, it was difficult to draw a clear line between the inclusion and exclusion of studies from the adjoining or overarching field of spousal support research. Including these was beyond the scope of this review. However, both research areas are intertwined and could enrich each other. Similarly, integrating studies on mental disease was beyond the scope of the current review.

Additionally, it was not possible to include studies that analyzed DC when both partners are affected by disease (even different ones). This could be another future research direction. Furthermore, we included only studies in which both partners' perspectives were taken into account.

Because of the broad range of studies included, we needed to apply various quality assessment tools, which varied in rigor and cannot easily be compared with each other. It is also important to consider the limitations of existing tools for assessing risk of reporting biases, in terms of their scope, guidance for reaching risk of bias judgments, and measurement properties (Page et al., 2018). We thus refrained from placing too much emphasis on individual study quality. To reduce the risk of publication bias, several authors were contacted directly to establish whether they had more studies in our field of interest. Further, it is important to note that we did not find any study without any significant results. Because in our narrative review we did we not collate data statistically, risk of selective reporting bias has not been assessed.

Finally, we focused only on DC in intimate relationships. Even though it is commendable that the literature increasingly views chronic disease as a joint coping effort of the person affected and their romantic partner, considering further significant others beyond the couple, such as adult children, parents, friends, extended family, or “fictive kin,” would be of merit (Taylor et al., 2013). Additionally, future research should consider intersectionality, which refers to the idea that we need to address diversity within social categories, hierarchies of privilege and power that structure social and material life, and commonalities across categories commonly viewed as profoundly different in psychological research (Cole, 2009).

### Conclusions

Facing challenges together as a couple seems vital for adapting to a diverse range of demands related to chronic physical diseases. Integrating a systemic focus should be a continuing effort in research, policy, and clinical practice in order to optimize medical, psychological, and psychosocial care in the current times of growing prevalence rates of chronic physical diseases across the globe. In the healthcare context, the family context needs to be taken into consideration, in particular the couple's relationship as a crucial aspect of physical and psychological recovery following a diagnosis. For research, implications point toward more longitudinal studies with diverse samples beyond the Global North. Lastly, we hope the review will stimulate the development of truly dyadic interventions with an eye on the specific challenges of the various chronic conditions. In the clinical context, findings point to the necessity of addressing both partners and taking both of them into account in interventions, as both are affected by the disease and suffer in their own way but also together. On the other hand, both partners (despite the disease and associated limitations and deficits) have shared resources that can be discovered and strengthened (Leuchtmann and Bodenmann, 2017). We believe that strengthening couples' resources (such as DC) in the context of the different diseases might be a promising direction for two reasons: (1) we can guarantee a better relationship functioning that is linked to better well-being and faster recovery from illness (Kiecolt-Glaser and Newton, 2001), and (2) by involving both partners in the intervention so that both feel considered, cared about, and important to the process of recovery or enduring the burden associated with the illness (Leuchtmann and Bodenmann, 2017).

## Data Availability Statement

The original contributions presented in the study are included in the article/Supplementary Material, further inquiries can be directed to the corresponding author.

## Author Contributions

GB and KW contributed to conception and design of the review. KW and FF organized the database. KW, FF, MR, and SL performed the data extraction. KW wrote the first draft of the manuscript. FF, MR, SL, and GB wrote sections of the manuscript. All authors contributed to manuscript revision, read, and approved the submitted version.

## Conflict of Interest

The authors declare that the research was conducted in the absence of any commercial or financial relationships that could be construed as a potential conflict of interest.

## Publisher's Note

All claims expressed in this article are solely those of the authors and do not necessarily represent those of their affiliated organizations, or those of the publisher, the editors and the reviewers. Any product that may be evaluated in this article, or claim that may be made by its manufacturer, is not guaranteed or endorsed by the publisher.
