# Supplementary material for: Dyadic Coping in Couples Facing Chronic Physical Illness: A Systematic Review
Source: Front Psychol. 2021 Oct 25;12:722740. doi: 10.3389/fpsyg.2021.722740 (PMC8573212; doi:10.3389/fpsyg.2021.722740)
Supplement: Supplementary file 1 [file Table_1.docx]

Supplement Table S1

*Summary of findings of quantitative studies*

| **Study** | **Design** | **Sample** | **DC Measures*** | **Non-DC Measures*** | **Main Findings** |
| --- | --- | --- | --- | --- | --- |
| **Rheumatoid Arthritis (RA) and lupus erythematosus** | | | | | |
| Dowdy, 1999 | Correlational non-experimental design | 80 US women with RA and their partners (age: M = 53 for patients and 52 for partners) | IV: Relationship Focused Coping Scale (RFCS, Coyne & Smith, 1991); Synchronous support (Revenson & Schiaffino, 1989) | Equity scale (Goodenow et al., 1990); General investment model (Rusbult, 1983); Perceived helpfulness of spouse (ad hoc); RA pain reduction | Support synchrony between the husband and wife did not reduce pain but it was shown to have a positive relationship to the wife's perception of her husband's helpfulness. The wife's rating of her husband's helpfulness was associated with being in a good marriage. The relationship between congruent coping and the husband's helpfulness was complex. |
| Fekete, Stephens, Mickelson & Druley, 2007 | Cross-sectional study | 243 US women with systemic lupus erythematosus and their partners (age: M = 39.4 for patients and 42 for partners) | Med.: Emotional support scale adapted from Stephens & Clark, 1997; Perceived Emotional Responsiveness (ad hoc) | CES-D; DAS | More spousal emotional support was interpreted as being more emotionally responsive, which in turn was related to better well-being. More problematic support was related to poorer well-being. When partners’ support efforts were responsive to the emotional needs, individuals also experienced fewer depressive symptoms and were more satisfied with their marriage. |
| Sterba et al., 2007 | Development and psychometric analysis of a new measure | 190 US women with RA and their partners (age: M = 49 for patients and 51 for partners) | Dyadic Efficacy for Married Women with Rheumatoid Arthritis; Perceptions of support over the past month (ad hoc) | Arthritis Impact Measurement Scale (Meenan et al., 1992); Arthritis understanding (ad hoc); Center for Epidemiologic Depression Scale (CES-D, Radloff, 1977); Health Survey Short Form (Ware et al., 1996); Kansas Marital Scale (Schumm et al., 1986); Optimism (Scheier et al., 1985); Positive and negative affect scale (PANAS, Watson et al., 1988); Quality of Marriage Index (QMI, Norton, 1983); Satisfaction With Life Scale (Diener et al., 1985); Teamwork standards (ad hoc) | The Dyadic Efficacy for Married Women with Rheumatoid Arthritis was developed with three subscales: arthritis problem solving and emotions, arthritis symptom management, and arthritis-related couple outcomes. It is a reliable instrument in parallel versions for patients and spouses. |
| **Chronic Obstructive Pulmonary Disease (COPD)** | | | | | |
| Meier, Bodenmann, Mörgeli & Jenewein, 2011 | Cross-sectional study | 43 Swiss patients with COPD and their partners (age: M = 68.2 for patients and 66.6 for partners) | DV: Dyadic Coping Inventory (DCI, Bodenmann, 2008) | Hospital Anxiety and Depression Scale (Zigmond & Snaith, 1983); Lung function Forced Expiratory Volume in 1 second (FEV1); World Health Organization Quality of Life Questionnaire (WHOQOL-BREF, Angermeyer et al., 2000) | Delegated coping was higher among partners than patients. In patients, high delegated partner coping and low delegated personal coping were associated with poorer quality of life for both patient and partner. Patients with additional depressive symptoms were supported more often and attributed deficits in dyadic coping primarily to themselves. Partners with additional depressive symptoms reported more own and partner negative coping. Patient perceived imbalance in delegated DC was associated with lower couple’s quality of life. |
| Meier et al., 2012  (same sample as Meier et al., 2011) | Cross-sectional study | 43 Swiss patients with COPD, their partners and 138 dyads as comparison group (age: M = 68.2 for patients, 66.6 for partners and 68.1 for comparison group) | DV: DCI | Lung function Forced Expiratory Volume in 1 second (FEV1) | COPD patients and their partners reported that the patients received more support and were less able to provide support to their partners. This difference was also evident in comparison with a community sample. Couples with COPD reported higher levels of negative coping and less positive dyadic coping. The dyadic coping of couples with COPD is unbalanced and more negative when compared to that of healthy couples. |
| Vaske et al., 2015 | Longitudinal study, 3 year follow up | 143 German COPD patients and their partners (age: M = 64.8 for patients and 62.1 for partners) | IV: DCI | European Quality of Life Questionnaire (EuroQol, EuroQol Group, 1990); Shuttle walk test (SWT, Singh et al., 1992); Symptom Cheklist (SCL-90-R, Franke, 1995) | Patients’ delegated and common DC rated by the spouses decreased over time. Patients’ quality of life at follow-up was positively influenced by partners’ stress communication. Partners’ quality of life at follow-up was negatively influenced by patients’ negative DC and positively influenced by partners’ delegated DC rated by patients. |
| **Diabetes mellitus** | | | | | |
| Berg et al., 2020  (same sample as Helgeson et al., 2019 & Lee et al., 2020) | Longitudinal diary study | 199 US patients with type 1 diabetes and their partners (age: M = 46.8 for patients and 46.4 for partners) | IV: Daily collaboration and support (ad hoc); Daily perceived coping effectiveness (ad hoc) | Couples Satisfaction Index (CSI-16, Funk & Rogge, 2007); Daily Illness Appraisal (ad hoc); Daily mean blood glucose (OneTouch Verio IQ glucometers, Malvern, PA); Daily self-care behaviors (Berg et al., 2014); Daily self-regulation failures (Berg et al., 2014); Hemoglobin A1c (HbA1c) | Illness appraisals were linked to collaboration and support strategies (C&S) both at the daily level and on average. On days when patients viewed diabetes as more shared than their own average, they reported fewer self-regulation failures. On days when patients viewed greater C&S than their own average, they reported more self-regulation failures. Patients who reported greater shared appraisal relative to the average of the sample reported better self-care. Greater shared appraisal was associated with higher self-care. Greater C&S strategies were associated with lower self-care. |
| Helgeson, 2017 | Cross-sectional study | 68 young adults from the US with type 1 diabetes (age: M = 25.52) | IV: Communal coping (ad hoc); Diabetes Family Behavior Checklist (Schaefer et al., 1986); Emotional support scale (Fekete et al., 2007); Overprotection Scale (Hagedoorn et al., 2000); Supportive and Unsupportive Interactions (ad hoc) | CES-D; Perceived Stress Scale (Cohen et al., 1983); Personal Assessment of Intimate Relationships (PAIR, Schaefer et al., 1981); QMI; Satisfaction with Life Scale | Partners were somewhat involved in diabetes management, but communal coping was less common compared to other chronically ill populations. The most common partner supportive behaviors were emotional and instrumental support and the most common partner unsupportive behavior was worry about diabetes. Communal coping was related to greater partner emotional and instrumental support, but also to greater partner overprotective and controlling behaviors. Partner overinvolvement in diabetes management had a mixed relation to outcomes, whereas partner under involvement was uniformly related to poor outcomes. |
| Helgeson et al., 2019  (same sample as Berg et al., 2020 & Lee et al., 2020) | Cross-sectional study | 199 US patients with type 1 diabetes and their partners (age: M = 46.8 for patients and 46.4 for partners) | IV: Collaborative, supportive and unsupportive behaviors (Berg et al., 2011; Helgeson et al., 2016); Illness appraisal (Zajdel et al., 2016) | CES-D; CSI-16; Diabetes Distress Scale (DDS, Polonsky et al., 2005); Hemoglobin A1c (HbA1c; DCA Vantage); Self Care Inventory (Lewin et al., 2009); SF-12 (Ware et al., 1993) | Partners were more likely than patients to hold shared illness appraisals. Patients’ shared appraisals were associated with more collaborative, instrumental, and emotional support, less protective buffering and more overprotective behavior. When patients and partners were consistent in their shared appraisals, support was highest. Collaborative, instrumental, and emotional support were related to better psychological and physical health when patients held shared compared to individual illness appraisals. |
| Helgeson, Jakubiak, Seltman, Hausmann & Korytkowski, 2017  (Subsample of Helgeson et al., 2020) | Cross-sectional study | 70 US patients with type 2 diabetes and their partners (age: M = 54.6 for patients and 55.6 for partners) | IV: Explicit communal coping (modified Inclusion of Other in the Self scale (IOS; Aron et al., 1992); Implicit communal coping (first person plural pronoun use); Overprotection Scale | CES-D; Diabetes Family Behavior Checklist; Emotional social support; Illness avoidance (Fekete et al., 2007); PAIR; Perceived Stress Scale; QMI; Satisfaction with Life Scale; Summary of Diabetes Self-Care Activities (SDSCA, Toobert & Glasgow, 1994) | Patient explicit communal coping was related to better patient relationship quality and greater support receipt from partners. Patient and partner explicit communal coping were related to reduced partner distress but not patient distress. Partner implicit communal coping was related to reduced patient distress and to better patient self-care behavior. Patients who reported greater overlap with their partners in coping with diabetes saw partners as more overprotective and controlling. There may be a limit on how much communal coping is adaptive. |
| Helgeson et al., 2020 | Cross-sectional study | 200 US patients with type 2 diabetes and their partners (age: M = 53.4 for patients and 53.2 for partners) | DV/IV: Behavioral coding of communal coping | Behavioral coding of warmth and hostility; CES-D; Diabetes issues (Feeney & Cassidy, 2003); Discussion evaluation, progress in resolving; Life Satisfaction Scale; PANAS; Perceived Stress Scale; QMI | Own and partner communal coping were related to good relationship and psychological functioning and higher levels of warmth. Partner communal coping was more beneficial for patients than spouses. Own communal coping was more beneficial for men, whereas partner communal coping was more beneficial for women. White patients and Black spouses benefitted more from own communal coping than Black patients and White spouses. |
| Helgeson, Seltman, Korytkowski & Hausmann, 2020  (Subsample of Helgeson et al., 2020) | Cross-sectional study | 123 US patients with type 2 diabetes and their partners (age: M = 54.2 for patients and 54.1 for partners) | IV: Behavioral coding of communal coping; Partner overprotective behavior (Hagedoorn et al., 2000); Patient perceived partner emotional responsiveness (ad hoc) | CES-D; Life Satisfaction Scale; Multidimensional Diabetes Questionnaire (Talbot et al., 1997); PAIR; Partner unmitigated communion (Helgeson & Fritz, 1998); Perceived Stress Scale; QMI; SDSCA | Partner communal coping was related to lower patient distress, higher patient self-efficacy, and higher patient medication adherence only when partners scored lower on unmitigated communion. |
| Helgeson & Van Vleet, 2019  (Subsample of Helgeson et al., 2020) | Cross-sectional study | 85 US patients with type 2 diabetes and their partners (age: M = 53 for patients and partners) | IV: Behavioral coding of communal coping; communal coping (Helgeson et al., 2018); IOS communal coping measure (IOS-CC); Use of "we-language" | IOS; QMI | Inclusion of other in self (IOS) was related to communal coping and relationship quality. |
| Hemphill, 2015 | Longitudinal diary study | 129 US patients with type 2 diabetes and their partners (age: M = 66.3 for patients and partners) | IV: Collaborative problem-solving among marital partners (Berg et al., 2003); Dyadic appraisal of responsibility for diabetes management (ad hoc) | Blood glucose; Diabetes Impact Management Scale (Hammond & Aoki, 1992); mood (ad hoc); Problem Areas in Diabetes Scale (PAID, Polonsky et al., 1995); QMI; Relationship quality (Prager & Buhrmester, 1998); SDSCA (Toobert et al., 2000) | Collaboration was not directly related to better emotional well-being for patients or spouses or patients’ disease management outcomes. Spouse collaboration did not significantly interact with dyadic appraisal to predict spouses’ relationship enjoyment, but patient collaboration did. |
| Johnson et al., 2013ᵃ | Cross-sectional study | 117 US patients with type 2 diabetes and their partners (age: M = 57.4 for patients and partners) | IV: DCI | Charlson comorbidity index (Charlson et al., 1987); CSI-4 (Funk & Rogge, 2007); Multidimensional Diabetes Questionnaire; SDSCA | Common dyadic coping was associated with higher levels of diabetes efficacy for both patients and spouses which, in turn, was then associated with better dietary and exercise adherence for the patient. |
| Johnson et al., 2013ᵇ  (same sample as Johnson et al., 2013ᵃ, 2015 & Trump et al., 2018) | Cross-sectional study | 117 US patients with type 2 diabetes and their partners (age: M = 57.4 for patients and partners) | DV: Ways of Giving Support (Buunk et al., 1996) | Charlson comorbidity index; Confidence Scale (Whitton et al., 2007); CSI-4; Marital Instability Over the Life Course (Amato & Booth, 1997); Multidimensional Diabetes Questionnaire; Satisfaction with Sacrifice Scale (Stanley & Markman, 1992); PAID; Revised Illness Perception Questionnaire (Moss-Morris et al., 2002); | A model was tested where the association of relationship satisfaction and active engagement was significantly stronger for men, while diabetes control was more strongly related to protective buffering for women. Active engagement was related to higher levels of relationship satisfaction (for men only), satisfaction with sacrifice, and relationship confidence. The diabetes appraisals were the only variables associated with protective buffering and overprotection. Higher diabetes distress and diabetes control (for women only) and lower diabetes efficacy were predictive of greater protective buffering. Lower diabetes efficacy and higher diabetes control were associated with greater overprotection. |
| Johnson et al., 2015  (same sample as Johnson et al., 2013ᵃ, 2013ᵇ & Trump et al., 2018) | Cross-sectional study | 117 US patients with type 2 diabetes and their partners (age: M = 57.4 for patients and partners) | IV: Ways of Giving Support | CSI-4; PAID; SDSCA | Spousal overprotection was indirectly associated with worse dietary adherence in patients with type 2 diabetes only when spouse active engagement was low. |
| Lee et al., 2020  (same sample as Berg et al., 2020 and Helgeson et al., 2019) | Cross-sectional study | 199 US patients with type 1 diabetes and their partners (age: M = 46.8 for patients and 46.4 for partners) | IV: Coping interview: proportion of first-person plural pronouns | Couples Satisfaction Index; Glycosylated hemoglobin (HbA1c, DCA Vantage Analyzer); Multidimensional Diabetes Questionnaire; Perceived Stress Scale; Spouse’s involvement in diabetes (ad hoc) | Greater spouse we-talk was associated with higher relationship satisfaction for both, higher patient self-efficacy and patient self-care, lower spouse stress and fewer spouse depressive symptoms. |
| Schokker et al., 2010 | Longitudinal study | 205 Dutch patients with type 1 or 2 diabetes and their partners (age: M = 54 for patients and partners) | IV/Mod.:Ways of Giving Support | Maudsley Marital Questionnaire (Arrindell et al., 1983) | Relationship satisfaction was positively associated with received active engagement and negatively with received protective buffering, in both patients and partners. Active engagement moderated the negative association between protective buffering and relationship satisfaction. Received protective buffering was negatively associated with relationship satisfaction, but only in patients who reported relatively low levels of received active engagement. |
| Trump et al., 2018  (same sample as Johnson et al., 2013ᵃ, 2013ᵇ, 2015) | Cross-sectional study | 117 US patients with type 2 diabetes and their partners (age: M = 57.4 for patients and partners) | Mod.: DCI | Acute Stress subscale of the Couple Stress Index (Bodenmann et al., 2008); Patient Health Questionnaire (PHQ-9; Kroenke et al., 2001); SDSCA | Spouse reported DC moderated the direct paths between spouses’ depression symptoms and patients’ adherence to dietary regimens, as well as the direct path between spouses’ acute stress and patients’ adherence to dietary regimens. |
| Van Vleet, Helgeson, Seltman, Korytkowski & Hausmann, 2018  (subsample of Helgeson et al., 2020) | Cross-sectional study | 119 US patients with type 2 diabetes and their partners (age: M = 54.5 for patients and 54.4 for partners) | DV/IV: Behavioral coding of communal coping | CES-D; Diabetes problem-solving (ad hoc); PANAS; QMI | More partner communal coping was linked with better perceived diabetes problem-solving, more positive mood, and less negative affect for females but not for males, and better relationship satisfaction for both males and females. |
| Van Vleet, Helgeson, Seltman, Korytkowski & Hausmann, 2019  (subsample of Helgeson et al., 2020) | Longitudinal study | 123 US patients with type 2 diabetes and their partners (age: M = 54 for patients and partners) | IV: Behavioral coding of communal coping, spouse emotional support provision, spouse instrumental support provision, and patient receptivity to spouse support | Diabetes Distress Scale; Multidimensional Diabetes Questionnaire; Perceived problem resolution (ad hoc); Perceptions of discussion (Feeney & Cassidy, 2003); SDSCA | Spouse communal coping was related to greater spouse emotional support and greater patient receptivity to spouse support. Communal coping was significantly linked to greater perceived problem resolution and more positive perceptions of the discussion. Perceived problem resolution and positive perception of the discussion mediated the link from patient communal coping to increases in self-efficacy and the link to decreases in diabetes distress. |
| Van Vleet & Helgeson, 2019  (subsample of Helgeson et al., 2020) | Longitudinal study | 86 US patients with type 2 diabetes and their partners (age: M = 55.4 for patients and partners) | IV/Mod.: Behavioral coding of communal coping | CES-D; Diabetes Distress Scale; Experiences in Close Relationships Questionnaire (Brennan et al., 1998); glycemic control; Multidimensional Diabetes Questionnaire; QMI; SDSCA | More avoidant patients engaged in less communal coping. Communal coping was related to positive benefits when patients were low in avoidance. When patients were high in avoidance, communal coping was either unrelated to outcomes or associated with poorer outcomes. |
| Zajdel, Helgeson, Seltman, Korytkowski & Hausmann, 2018  (subsample of Helgeson et al., 2020) | Longitudinal diary study | 123 US patients with type 2 diabetes and their partners (age: M age = 54 for patients and partners) | IV: Communal coping (ad hoc); Perceived Emotional Responsiveness (Fekete et al., 2007) | Profile for Mood States (Usala & Hertzog, 1989); Self-care behaviors (ad hoc) | Actor communal coping was associated with lower depressed mood, higher happy mood, and lower angry mood. Partner communal coping was linked to higher happy mood. Patient communal coping was related to better dietary and medication adherence. Spouse communal coping was linked to better medication adherence. |
| **Renal Disease** | | | | | |
| Tkachenko, Franke, Peters, Schiffer & Zimmermann, 2019 | Cross-sectional study | 56 German patients with renal disease and their partners (age: M = 58.1 for patients and 57.2 for partners) | DV: DCI | General Anxiety Disorder Screener (GAD-7, Spitzer et al., 2006); Patient Health Questionnaire; Quality of Marriage Index German version (QMI-D, Zimmermann et al., 2015) | In couples with male patients, female caregivers showed more own supportive DC than the males. In couples with female patients, women reported higher own stress communication, supportive DC, total positive DC and total DC as well as depression compared to men. Low DC discrepancies were associated with positive psychological outcomes. |

*Note*: * In some cases only a selection of items was used instead of the complete scales. DV = dependent variable, IV = independent variable, med. = mediator, mod. = moderator
