# Supplementary material for: Dyadic Coping in Couples Facing Chronic Physical Illness: A Systematic Review
Source: Front Psychol. 2021 Oct 25;12:722740. doi: 10.3389/fpsyg.2021.722740 (PMC8573212; doi:10.3389/fpsyg.2021.722740)
Supplement: Supplementary file 2 [file Table_2.docx]

Supplement Table S2

*Summary of findings of qualitative studies*

| **Study** | **Design** | **Sample** | **Data Collection** | **Data Analysis** | **Main Findings** |
| --- | --- | --- | --- | --- | --- |
| **Arthritis and lupus erythematosus** | | | | | |
| Mann & Dieppe, 2006 | Qualitative interview study | 12 UK patients with RA and their partners (age: M = 50 for women with RA, 60 for men with RA, partners age: range 29-72) | Semi-structured interviews, couples together | Interpretative phenomenological analysis | Couples were allocated to one of three groups: the shared illness management group, the ill partner in charge group, or the conflict over management group. The last group consisted only of couples with men with RA. |
| Yorgason et al., 2010 | Qualitative interview study | 28 US patients with OA and diabetes type 2 and their partners (age: M = 68 for patients and partners) | Semi-structured interviews, couples together | Qualitative content analysis, methodological approach, grounded theory | Couples differed in terms of how they handled the illness: some couples seemed to handle problems as individuals, some appeared to cope in a fully shared nature, and others had a mix of individual and shared coping activities. Positive ways of coping were often an emphasis on teamwork and sharing the responsibility of illness. This included shared decision making about meals, exercise, and overall schedule. Couples also solved problems in collaborative ways to make necessary changes to cope more effectively with diabetes and osteoarthritis. |
| **Cystic Fibrosis** | | | | | |
| Werner, Hochman, Rosenne & Kurtz, 2020 | Qualitative interview study | 17 patients with cystic fibrosis from Israel and their partners (age: M = 38.1 for patients and partners) | Semi-structured interviews, couple separately | Interpretative phenomenological analysis | Cooperation and tension were two main patterns of DC with CF that were identified. Couples in cooperation shared a similar attitude regarding the place of the illness in their lives and the way it should be handled, responsibility of the CF patient for managing the illness, agreed-upon division of roles, optimism and hope, concentrating on present challenges, open communication, mutual empathy, instrumental and emotional support, and marital growth. Couples in tension were characterized by discrepancy in illness perception and the means they employed to cope with it, perceptions of the disease as a disruptive factor, having difficulty seeing each other’s needs and communicating about the disease, and providing each other with appropriate support. |
| **Diabetes mellitus** | | | | | |
| Beverly & Wray, 2010  (same sample as Beverly et al., 2008) | Qualitative interview study | 30 US patients with type 2 diabetes and their partners (age: M = 66.7 for patients and 63.3 for partners) | Focus group | Focus group analysis | Couples who felt they were 'in this together' were better able to communicate and thus support each other in their daily management. How couples interpreted their support may have informed and altered their motivation and responsibility to exercise. Spouses of persons with diabetes felt that they walked a fine line between encouraging and nagging their spouses to exercise. Women seemed to serve as gatekeepers to the family health and to be more practiced and better equipped to provide effective support to their spouses living with diabetes than vice versa. |
| Beverly, Miller & Wray, 2008  (same sample as Beverly & Wray, 2010) | Qualitative interview study | 30 US patients with type 2 diabetes and their partners (age: M = 66.7 for patients and 63.3 for partners) | Focus group | Focus group analysis | Couples that did talk about diabetes appeared to have greater spousal support and fewer difficulties with dietary adherence. Husbands who relied on their spouses to maintain a healthful diet exhibited lower self-control over their own dietary changes. Women diagnosed with diabetes perceived a lack of support from their husbands regarding dietary choices. Depending on who is suffering from diabetes in the relationship DC may be experienced differently, as a form of control (sensed by male patients) or lack of support (sensed by female patients). |
| Trief et al., 2003 | Qualitative interview study | 40 US patients with diabetes (55% type 2) and 32 partners (age: M = 49 for patients and partners) | Semi-structured interviews | Grounded theory | Difficulties were seen in coping with issues related to the independence of the patient and hypoglycemia. Regimen specific support, general relational support, support in dietary control and reminders were seen as helpful, whereas the line is thin between reminders and nagging, which was seen as not helpful, just as problems with diet management and poor communication. |
| **Endometriosis** | | | | | |
| Brown, 2007 | Qualitative interview study | 10 US women with dyspareunia due to endometriosis and their partners (age: range 20-42 for patients and partners) | Semi-structured interviews, couple together | Interpretative phenomenological analysis | Most women had developed endometriosis before entering the relationship. Women seemed to be the ones getting the information and sharing it with men, still they felt that they were coping as a team. The women stressed how important it was to talk about the illness as well as the relationship. The partners came to understand their partners’ cycles, moods, and triggers over time. Communication about the dyspareunia seemed particularly important in adapting their intimate relationship to avoid pain. |
| **HIV** | | | | | |
| Montgomery, Watts & Pool, 2012 | Qualitative interview study | 10 HIV-serodiscordant couples from Uganda and Zambia | Semi-structured interviews, couple together | Thematic content analysis | In Ugandan serodiscordant couples with men suffering from HIV, couple HIV testing and joint seminars were a key factor in spouses’ (especially male spouses’) transformation of motivation. Both couple members believed that joint effort was needed and of advantage. They communicated about the situation and engaged in cooperative action to solve problems including use of condoms and gel. In the Zambian sample, protecting the health of the HIV-negative woman was left to the women. Communication occurred when permission was being sought by the woman for her behavior and, as suggested by some, against the tacit threat of marital conflict if they did not. |
| Rispel, Cloete, Metcalf, Moody & Caswell, 2012 | Qualitative interview study | 36 HIV-serodiscordant couples from South Africa and Tanzania (age: range = 20 – 54) | Semi-structured interviews, individually and couple together | Thematic content analysis | Three communication patterns were described. ‘Sero-sharing’ occured in couples who viewed HIV as being an issue which they dealt with together. The coping style coined as ‘sero-normalcy’ related to partners who saw their relationship as ‘normal’ or not different from other couples not affected by HIV. Yet, HIV still seemed to be a factor that affected their relationship. ‘'Sero-difference’ referred to couples who experienced their relationship as being different from other couples’ relationships, because of it being overshadowed by the experience of living with HIV in particular when dealing with safer sex or their desire to having children. Some couples exhibited features of both coping styles and, at times, partners differed in their ways of coping. |
| **Parkinson's Disease** | | | | | |
| Martin, 2014 | Qualitative interview study | 21 US patients with Parkinson’s disease and their partners (age: M = 68 for patients and 67 for partners) | -Semi structured interviews | Grounded theory | Emotional support, listening like one partner described, “He’ll say, ‘I don’t like what’s happening to me.’ And I listen” (Martin, 2014, S. 142). Providing informational support, giving advice, encouraging perspective shifts. However, participants described, partners’ differing approaches to coping can make support difficult or support may threaten the independent and capable identity or place unwanted emphasis on the disease. Support can also be interpreted as controlling. Fear of burdening the other and actual draining of the caregiver. Dealing with dependency Framing partners as equal, relinquishing control, humor, seeking/providing support indirectly or subtly, taking the other’s perspective. |
| **Renal Disease** | | | | | |
| Wise, Schatell, Klicko, Burdan & Showers, 2012 | Qualitative interview study | 13 US patients with renal disease and their partners (age: M = 57.1 for patients and 56.5 for partners) | In depth semi-structured telephone interviews | Grounded theory | Four profiles or DC were described: (1) Thriving —patients and care partners were flourishing; (2) Surviving—strong couples were adjusting to challenges; (3) Martyrdom—one partner defers his/her needs and resentments to make home dialysis work; and (4) Seeking another option—patient unwilling to burden an anxious partner. Dialysis training that was unhurried and valued care partners as well as patients, used a mix of learning strategies, and provided a home visit for the first home treatment was associated with the group of Thriving dyads. |
| **Stroke** | | | | | |
| Robinson-Smith & Mahoney, 1995 | Qualitative interview study | 7 US stroke survivors and their partners (age: range = 60 – 82 for patients and partners) | Semi-structured interviews | Content analysis | Couples talked about how a new equilibrium was being sought together in an attempt to resolve the impact of the stroke and adapt to changes in formerly shared leisure activities, or division of homemaking and breadwinning labor. Stroke spouses often took over more household work to support the stroke survivor. |
