# Supplementary material for: Dyadic Coping in Couples Facing Chronic Physical Illness: A Systematic Review
Source: Front Psychol. 2021 Oct 25;12:722740. doi: 10.3389/fpsyg.2021.722740 (PMC8573212; doi:10.3389/fpsyg.2021.722740)
Supplement: Supplementary file 3 [file Table_3.docx]

Supplement Table S3

*Summary of findings of mixed methods studies*

| **Study** | **Design** | **Sample** | **DC Measures** | **Qualitative Data Collection and Analysis** | **Main Findings** |
| --- | --- | --- | --- | --- | --- |
| **HIV** | | | | | |
| (Ⅰ) Gamarel, 2014ᵃ  (Ⅱ) Gamarel, Comfort, Wood, Neilands & Johnson, 2016ᵃ | (Ⅰ) Longitudinal study, 24-month follow-up  (Ⅱ) Qualitative interview study | (Ⅰ) 117 HIV-positive US men and their partners (age: M = 46.6)  (Ⅱ) 20 HIV-positive US men and their partners (age: M = 50.6) | (Ⅰ) Communication Patterns Questionnaire Short Form (Christensen & Heavey, 1990); Inclusion of Other in Self scale (IOS, Aron et al., 1992) | (Ⅱ) Semi structured interview; IOS Diagram  Framework analysis | (Ⅰ) Positive communication was associated with increased relationship satisfaction and more negative communication was associated with decreased relationship satisfaction for both partners. HIV-negative partners’ negative communication was associated with lower levels of non-adherence and anal sex. Both partners’ positive communication fully mediated the relationship between their own IOS and relationship satisfaction. Partners who report higher levels of IOS at baseline tended to report higher levels of positive communication at 6-months, and higher levels of relationship satisfaction at 12-months.  (Ⅱ) Couples described two basic orientations toward health: ‘Relational’ and 'Personal'. Couples who held relational orientations described their health as interconnected and prioritized being aware of each other's health status and care needs. Within this group, a subgroup was termed ‘Asymmetrical’, describing couples in which one partner's health was prioritized over the other’s. The personal orientations group consisted of couples in which one or both partners described their health and health care as independent and autonomous. However, this group also indicated that this was changeable depending on their partner’s health status. Couples who held more autonomous orientations to their own and their partners’ health also were satisfied with their partners’ support strategies. |
| **Stroke** | | | | | |
| McCarthy, 2012 | Mixed methods | 32 US stroke survivors and their partners (age: M = 62.1 for patients and 60.7 for partners) | Ways of Giving Support (Buunk et al., 1996) | Semi structured phone interview; constant comparative method | Lower levels of active engagement and higher levels of protective buffering were associated with greater depression in spouses but not in patients. Survivors who perceived greater understanding from spouses reciprocated by actively engaging with their spouse around their personal experience with the illness. Qualitative analysis yielded relationship challenges, like shifting from pre- to post-stroke roles. Some couples talked about how the illness had brought them closer together. |

*Note:* ᵃ Subsample of: The Duo Project, Conroy et al., 2016

Conroy, A.A., Gamarel, K.E., Neilands, T.B. et al. Relationship Dynamics and Partner Beliefs About Viral Suppression: A Longitudinal Study of Male Couples Living with HIV/AIDS (The Duo Project). *AIDS Behavior 20,*1572–1583 (2016). https://doi.org/10.1007/s10461-016-1423-9
