# Supplementary material for: Dyadic Coping in Couples Facing Chronic Physical Illness: A Systematic Review
Source: Front Psychol. 2021 Oct 25;12:722740. doi: 10.3389/fpsyg.2021.722740 (PMC8573212; doi:10.3389/fpsyg.2021.722740)
Supplement: Supplementary file 4 [file Table_4.docx]

Supplement Table S4

*Summary of findings of intervention studies*

| **Study** | **Design** | **Sample** | **Measures** | **Intervention approach & format** | **Control Type** | **Main Findings** |
| --- | --- | --- | --- | --- | --- | --- |
| **Arthritis and lupus erythematosus** | | | | | | |
| Keefe et al., 1996 | Randomized controlled trial (RCT)  Pre- & post intervention | 88 US patients with OA and their partners (age: M = 62.6 for patients and partners) | Arthritis Impact Measurement Scales (AIMS, Meenan et al., 1980); Arthritis Self-Efficacy Scale (Parker et al., 1995); Coping Strategies Questionnaire (Rosenstiel & Keefe, 1983); Dyadic Adjustment Scale (DAS, Spanier, 1986); Pain behavior (behavioral coding); Medication use | Spouse assisted cognitive behavioral coping skills training (SA-CST)  10 weekly 2-hour group sessions | Arthritis education-spousal support (AE-SS) or individual coping skills training (CST) | Spouse-assisted CST proved to be more effective than an arthritis education intervention with spouse involvement (control condition). The patients in the spouse-assisted CST condition showed significantly lower post-treatment levels of pain and psychological disability and showed less post-treatment pain behavior than patients in the control condition. Patients in the spouse-assisted CST condition reported significantly higher self-efficacy and more frequent use of pain-coping strategies compared with patients in the control condition at post-treatment. |
| Keefe et al., 1999  (same sample as Keefe et al., 1996) | RCT  Pre-intervention, 6- & 12 months follow up | 88 US patients with OA and their partners (age: M = 62.6 for patients and partners) | AIMS; Arthritis Self-Efficacy Scale; Coping Strategies Questionnaire; DAS; Pain behavior (behavioral coding); Medication use | Spouse assisted cognitive behavioral coping skills training (SA-CST)  10 weekly 2-hour group sessions | Arthritis education-spousal support (AE-SS) or individual coping skills training (CST) | The spouse-assisted CST was found to enhance self-efficacy and improve coping abilities of OA patients in the long term. There were individual differences in the long-term outcome of spouse-assisted CST: those patients with increases in marital satisfaction and self-efficacy showed much better outcomes than others. |
| Keefe et al., 2004 | RCT  Pre- & post intervention | 72 US patients with OA and their partners (age: M = 59.5 for patients and partners) | Aerobic fitness and strength measures; AIMS; Arthritis Self-Efficacy Scale (Lorig et al., 1989); Arthritis Self-Efficacy Scale spouse version (Keefe et al., 1996a,b, 1997, 1999); Coping Strategies Questionnaire; DAS | Spouse assisted cognitive behavioral coping skills training (SA-CST) with or without exercise training  12 weekly 2-hour group sessions | Exercise training (ET) or standard care | Spouse-assisted training, either alone or in combination with exercise training, lead to improved coping and self-efficacy. Exercise training, either alone or in combination with spouse-assisted coping skills training improved physical fitness and muscle strength. |
| Van Lankveld, van Helmond, Näring, de Rooij & van der Hoogen, 2004 | Non-randomized controlled trial  Pre- & post- intervention, 2 weeks & 6 months follow up | 59 Dutch patients with RA and their partners (age: M = 49.5 for patients and partners) | Communication improvement (ad hoc); Coping with Rheumatoid Stressors (van Lankveld et al, 1992); Disease Activity Score (Prevoo et al., 1995); Erythrocyte sedimentation rate (ESR); Impact of Rheumatic Diseases on General Health and Lifestyle (Huiskes et al., 1990); Maudsley Marital Questionnaire (Arrindell et al., 1983); Spouse reaction questionnaire (Kraaimaat et al., 1995) | Spouse assisted training to restructure disease related cognitions and teach effective coping styles using rational emotive therapy (RET)  8 1.5-hour group sessions, twice a week | Group treatment program without partner | In both, intervention and control condition, similar positive changes in disease activity, cognitions, coping, and physical and psychological functioning were observed. Patients reported a decrease in potential support. At follow-up assessment, patients in the experimental condition reported higher improvement of disease related communication with their spouse compared with the control patients. |
| **Diabetes mellitus** | | | | | | |
| Trief, Sandberg, Ploutz-Snyder, Brittain & Cibula, 2011 | Pilot study, RCT  Pre- & post-intervention, 3- & 6 months follow up | 44 US patients with type 2 diabetes and their partners (age: M = 59.9 for patients) | Blood pressure, Glycemic control; Summary of Diabetes Self-Care Activities (SDSCA, Toobert & Glasgow, 1994); Total and LDL cholesterol; Waist circumference | Telephonic couple behavioral diabetes intervention  10 couple sessions via telephone | Individual phone intervention and individual diabetes education | Of the total three intervention groups studied, both individual and couple interventions showed meaningful clinical improvements in medical outcomes. The diabetes education intervention resulted in improved blood glucose control. Significant treatment effects for total cholesterol were found. |
| Trief et al., 2016 | RCT  Pre- & post-intervention, 8- & 12 months follow up | 280 US patients with type 2 diabetes and their partners (age: M = 56.8 for patients) | Blood pressure; Diabetes Distress Scale (Polonsky et al., 2005); Diabetes self-efficacy (Lorig et al., 2009); Glycemic control; Obesity; Patient Health Questionnaire (PHQ-8, Kroenke et al., 2009); Patient satisfaction questionnaire (ad hoc) | Telephonic couple behavioral diabetes intervention  12 couple sessions via telephone | Individual phone intervention and individual diabetes education | Significant A1C reductions were found in all study arms. For the primary outcome of glycemic control, there were no between group differences. Subgroup within-arm analyses showed that the CC intervention was efficacious in lowering A1C levels for high A1C level individuals. For BMI as outcome, CC showed significant improvement and CC and DE led to decreased waist circumference. |
| **Parkinson’s Disease** | | | | | | |
| Lyons et al., 2020 | Pilot study, quasi experimental design  Pre-intervention & 7 weeks follow up | 49 US patients with parkinson’s disease and their partners (age: M = 70.6 for patients, 68.4 for partners and 65.6 for control group) | Center for Epidemiologic Depression Scale (CES-D, Radloff, 1977); Multidimensional Caregiver Strain Index (Stull, 1996); Self-management variables (Lorig et al., 1996); SF-36 (Ware & Sherbourne, 1992); Ways of Giving Support (Buunk et al., 1996) | Chronic disease self-management program (CDSMP)  7 spouse assisted group sessions | Waitlist control group | Spouses in the Strive to Thrive self-management program group showed greater engagement in mental relaxation techniques after 7 weeks in comparison to spouses in the wait-list control condition. No changes were observed for active engagement. |
| **Stroke** | | | | | | |
| Robinson-Smith, Harmer, Sheeran & Vallo, 2016 | Pilot study, RCT  Pre- & post-intervention | 10 US stroke survivors and their partners (age: M = 65.2 for patients, 65.4 for partners and 75.6 for control group) | Activity Measure for Post-Acute Care (Jette et al., 2007); CES-D; Dyadic Coping Inventory (DCI, Bodenmann, 2008); Strategies used to promote peoples’ health (Lev & Owen, 1996); Quality of life index-stroke version (Ferrans & Powers, 1992) | Psychoeducational intervention (PEI) to expand coping skills  6 individual home visits | No treatment | Significant increases in coping by oneself and quality of life were found for stroke survivors in the experimental group, while their depressive symptoms decreased. A significant increase in positive dyadic coping occurred for spouses in the experimental group. |
| Terill et al., 2018 | Pilot study, RCT  Pre- & post-intervention, 3 months follow up | 11 US stroke survivors and their partners (age: M = 56.0 for patients and 55.9 for partners) | Acceptability and helpfulness of intervention (ad hoc); Connor Davidson Resilience Scale (Campbell-Sills & Stein, 2007); Older People’s Quality of Life Questionnaire (Bowling, 2009); PROMIS-Depression Short Form 8b (Cook et al., 2012); Social Relationships Index (Campo et al., 2009); Stroke Impact Scale (Vellone et al., 2015) | Positive psychology-based intervention (PPI)  self-administered 8-week intervention and three in-person study sessions | Waitlist control group | The self-administered dyadic positive psychology-based intervention (PPI) is feasible for implementation with couples poststroke. |
